# TRPM7 kinase activity is essential for T cell colonization and alloreactivity in the gut

**DOI:** 10.1038/s41467-017-01960-z

**Published:** 2017-12-04

**Authors:** Andrea Romagnani, Valentina Vettore, Tanja Rezzonico-Jost, Sarah Hampe, Elsa Rottoli, Wiebke Nadolni, Michela Perotti, Melanie A. Meier, Constanze Hermanns, Sheila Geiger, Gunther Wennemuth, Camilla Recordati, Masayuki Matsushita, Susanne Muehlich, Michele Proietti, Vladimir Chubanov, Thomas Gudermann, Fabio Grassi, Susanna Zierler

**Affiliations:** 1 Institute for Research in Biomedicine, Università della Svizzera Italiana, Via Vincenzo Vela 6, CH-6500 Bellinzona, Switzerland; 2 Graduate School for Cellular and Biomedical Sciences, University of Bern, c/o Theodor Kocher Institute, Freiestrasse 1, P.O. Box 938, CH-3000 Bern 9, Switzerland; 3 Walther Straub Institute of Pharmacology and Toxicology, Ludwig-Maximilians Universität München, Goethestrasse 33, 80336 Munich, Germany; 4 Department of Medical Biotechnology and Translational Medicine (BIOMETRA), Università degli Studi di Milano, Via G.B. Viotti 3/5, 20133 Milan, Italy; 5 Institute for Anatomy, Universitätsklinikum Essen, Hufelandstrasse 55, 45147 Essen, Germany; 6 Filarete Foundation, Viale Ortles 22/4, 20139 Milan, Italy; 7 Department of Molecular and Cellular Physiology, Graduate School of Medicine, University of the Ryukyus, 207 Uehara, Okinawa, 903-0215 Japan; 8 Istituto Nazionale Genetica Molecolare “Romeo ed Enrica Invernizzi”, Via Francesco Sforza, 35-20122 Milan, Italy; 9 Present Address: Center for Chronic Immunodeficiency, Universitätsklinikum Freiburg, Breisacher Street 115, 79106 Freiburg, Germany

## Abstract

The melastatin-like transient-receptor-potential-7 protein (TRPM7), harbouring a cation channel and a serine/threonine kinase, has been implicated in thymopoiesis and cytokine expression. Here we show, by analysing TRPM7 kinase-dead mutant (*Trpm7* ^*R/R*^) mice, that the enzymatic activity of the receptor is not essential for thymopoiesis, but is required for CD103 transcription and gut-homing of intra-epithelial lymphocytes. Defective T cell gut colonization reduces MHCII expression in intestinal epithelial cells. Mechanistically, TRPM7 kinase activity controls TGF-β-induced CD103 expression and pro-inflammatory T helper 17, but not regulatory T, cell differentiation by modulating SMAD2. Notably, we find that the TRPM7 kinase activity promotes gut colonization by alloreactive T cells in acute graft-versus-host disease. Thus, our results unravel a function of TRPM7 kinase in T cell activity and suggest a therapeutic potential of kinase inhibitors in averting acute graft-versus-host disease.

## Abstract (web summary)

Gut-homing and colonization of T cells are important for maintaining local immune homoeostasis and protective immunity. Here the authors show that the kinase activity of TRPM7 regulates Th17 differentiation and T cell alloreactivity in the gut by modulating SMAD2 activation and CD103 expression in T cells

## Introduction

The antigen-rich environment of the gut interrelates with a highly specialized mucosal immune system, mastering the challenge of preventing invasion and systemic spread of microbes while avoiding unnecessary immune reactions to commensal bacteria. Besides representing a physical barrier, the intestinal epithelium constitutes also a dynamic interface between the host immune system and the luminal environment, which harbours potentially harmful microbes. Therefore, maintenance of the protective barrier is essential in mucosal immunity, and intra-epithelial lymphocytes (IEL) have an important function in maintaining this barrier function^1^. The intestinal mucosa is composed of a single layer of columnar epithelial cells, the underlying lamina propria and the muscularis mucosa. Tight junctions, components of the apical junctional complex, seal the paracellular space between epithelial cells. IELs are located above the basement membrane, but are subjacent to tight junctions. The lamina propria is located beneath the basement membrane and contains immune cells, including macrophages, dendritic cells and lamina propria lymphocytes (LPL)^2^. Intestinal T cells are highly heterogeneous in phenotype and function and include both conventional and unconventional subpopulations. Conventional mucosal T cells express the αβ T cell receptor (TCRαβ) together with CD4 or CD8αβ as co-receptors, whereas unconventional mucosal T cells express either TCRαβ or TCRγδ together with CD8αα homodimers^1^. During their activation in specialized mesenteric lymph nodes or Peyer’s patches, naive T cells acquire gut-homing properties through the upregulation of distinct adhesion receptors including the integrins α_4_β_7_ and α_E_β_7_ (CD103)^3, 4^. Moreover, the resident microbiota regulates the development of specific lymphocyte subsets in the gut. CD4^+^ T helper 17 (T_H_17) cells preferentially accumulate in the intestine, indicating a developmental regulation by gut-intrinsic mechanisms^5^. Forkhead box P3 (FoxP3) expressing regulatory T (T_reg_) cells represent another CD4^+^ T helper (T_H_) cell subset that preferentially accumulates in the intestine and contributes to gut homoeostasis. The regulated induction of pro-inflammatory T_H_17 and immunosuppressive T_reg_ cells in the gut illustrates the importance of an equilibrium between effective immunity and tolerance to preserve tissue integrity^1^. However, the mechanisms responsible for this physiologic balance are not well understood. The induction of both these T_H_ subsets depends on TGF-β, which is abundantly present in the intestine^6, 7^.

Among the mammalian transient receptor potential (TRP) superfamily of unselective cation channels, the TRPM subfamily, named after its founding member melastatin, TRPM1^8^, comprises eight members including the dual-function protein, TRPM7. TRPM7 is a divalent selective cation channel, mainly conducting Mg^2+^, Ca^2+^ and Zn^2+^, fused to a C-terminal α-kinase domain^9, 10^. TRPM7 has been implicated in cell survival, proliferation, apoptosis as well as migration and immune cell function. However, the physiologic function of TRPM7 ion channel or enzymatic activity is poorly understood^11, 12^. Unlike conventional kinases, TRPM7 kinase does not recognize known specific amino acid motifs but phosphorylates serines (Ser) and threonines (Thr) located within alpha-helices^10^. TRPM7 contains a Ser/Thr-rich autophosphorylation site, which aids in TRPM7-substrate binding^13^. In vitro, TRPM7 kinase phosphorylates annexin A1^10, 14^, myosin II isoforms^15^, eEF2-k^16^ and PLCγ2^17^.

Deletion of the ubiquitously expressed TRPM7 protein is embryonic lethal^18, 19^. Deletion of the exons encoding only the TRPM7 kinase domain (*Trpm7* ^*ΔK/ΔK*^) also causes early embryonic death, most probably attributable to reduced channel function in this mutant^19^. However, heterozygous mice (*Trpm7* ^*+/ΔK*^) are viable and develop severe hypo-magnesaemia upon Mg^2+^ restriction, causing increased mortality, susceptibility to seizures and prevalence for allergic hypersensitivity^19^. Interestingly, homozygous mice with genetic inactivation of TRPM7 kinase activity by a point mutation within the active site of the kinase (K1646R, *Trpm7* ^*R/R*^) have no obvious phenotype^20, 21^, indicating that the *Trpm7* ^*+/ΔK*^ phenotype, is due to decrease in both channel and kinase activity. Moreover, analysis of these mouse models revealed that TRPM7 kinase activity regulates mast cell degranulation and histamine release, implicating TRPM7 in the hyper-allergic phenotype observed previously^22^. Tissue-specific deletion of *Trpm7* in the T cell lineage disrupts thymopoiesis and results in altered chemokine and cytokine expression profiles^18^, indicating that TRPM7 channel and/or kinase are important for T cell function.

Here we show that the ubiquitous kinase-dead mouse model, *Trpm7* ^*R/R*^, with a single point mutation at the active site of the kinase^21^ has an exquisite requirement for TRPM7 kinase activity in intra-epithelial T cell homoeostasis. We find that gut colonization by alloreactive T cells in acute graft-versus-host disease depends on TRPM7 kinase activity, indicating a therapeutic potential of kinase inhibitors in averting this condition.

## Results

### TRPM7 kinase does not affect channel activity

To investigate the impact of the TRPM7 kinase on T cell function, we utilized a mouse model carrying a point mutation at the active site of the enzyme^21^. Mutating lysine at position 1646 to arginine (*Trpm7* ^*R/R*^) disrupts ATP binding and thereby kinase activity (Supplementary Fig. 1a)^21^. Using immunoprecipitation and western blot analysis, we were able to confirm that the mutation indeed disrupted native kinase activity and thus autophosphorylation at serine 1511 in primary splenocytes (Supplementary Fig. 1b). Unlike mice lacking the entire kinase domain^19^, homozygous *Trpm7* ^*R/R*^ mice are viable^20, 21^. They are normal in size, weight and Mendelian inheritance ratio compared to wild-type (WT^)20, 21^. To test whether inactivation of TRPM7 kinase has any effect on Mg^2+^ and Ca^2+^ homoeostasis, we used inductively coupled mass spectrometry (ICP-MS), biochemical as well as calcium-imaging techniques. By ICP-MS, we observed no changes in serum Mg^2+^ and Ca^2+^ concentrations (Supplementary Fig. 1c, d). Cellular ATP levels are often taken as an estimate for intracellular Mg^2+^ contents^23^. Therefore, we performed a luciferin luciferase assay and found no alterations in intracellular ATP levels between WT and *Trpm7* ^*R/R*^ primary naive CD4^+^ T cells (Supplementary Fig. 1e). To determine basal intracellular free Ca^2+^ concentrations ([Ca^2+^]_i_), we used ratiometric Fura-Red imaging. No significant differences in [Ca^2+^]_i_ between WT and *Trpm7* ^*R/R*^ primary naive CD4^+^ T cells were detected (Supplementary Fig. 1f). Further, we assessed the potential function of kinase activity in the regulation of biophysical features of the TRPM7 channel. Whole-cell patch-clamp experiments revealed that the channel function is unaltered in primary peritoneal mast cells (Supplementary Fig. 1g, h) as well as in naive CD4^+^ T cells (Supplementary Fig. 1j), which is in line with previous reports on peritoneal macrophages and mast cells, as well as embryonic fibroblasts isolated from *Trpm7* ^*R/R*^ mice^20–22^. *Trpm7* ^*R/R*^ channels display slightly decreased Mg^2+^-sensitivity without obvious consequences for the channel activity at physiologic Mg^2+^ levels (Supplementary Fig. 1i). As already shown, serum Mg^2+^ and Ca^2+^ concentrations were unaffected (Supplementary Fig. 1c, d)^21^. This overall constellation allowed us to independently investigate TRPM7 kinase function.

### TRPM7 kinase affects serum cytokines but not thymopoiesis

**Fig. 1:**
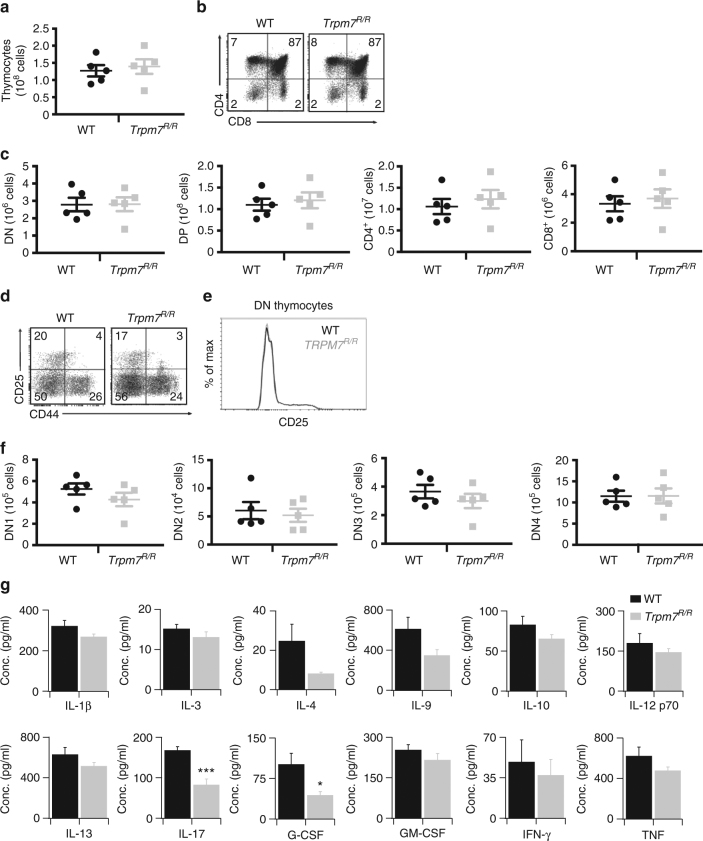
Normal T cell development in *Trpm7* ^*R/R*^ mice but altered cytokine secretion. **a** Total WT or *Trpm7* ^*R/R*^ cell recovery from thymus. **b** Representative dot plot analysis of thymocytes from WT or *Trpm7* ^*R/R*^ thymi stained with CD4 and CD8 mAbs. Percentages are shown in each gate. **c** Dot charts comparing the total number of thymocytes in the double-negative (DN), double-positive (DP), CD4^+^, and CD8^+^ thymocytes are shown (mean ± s.e.m. *n* = 5). **d** Representative dot plot analysis of thymocytes gated on DN cells from WT or *Trpm7* ^*R/R*^ thymi stained with CD44 and CD25 mAbs. Percentages are shown in each gate. **e** Representative histogram overlay of cell surface CD25 in WT or *Trpm7* ^*R/R*^ thymocytes. **f** Dot charts showing the number of total cells (mean ± s.e.m. *n* = 5) of DN population found in the DN1, DN2, DN3 and DN4 stages. Data are representative results of two independent experiments with five mice per experiment. **g** Basal cytokine levels evaluated in serum of WT (black, *n* = 3–7) and *Trpm7* ^*R/R*^ (grey, *n* = 3–7) mice, respectively, and shown as pg ml^−1^. Bar charts indicate mean ± s.e.m. A total number of seven mice were used for each genotype. Note a significant reduction of serum levels of IL-17 and G-CSF in *Trpm7* ^*R/R*^. A two-tailed Student’s *t* test was used with *p < 0.05; **p < 0.01 and ***p < 0.001

Tissue-specific deletion of *Trpm7* in the T cell lineage was shown to disrupt thymopoiesis and resulted in altered chemokine and cytokine expression profiles^18^, indicating that TRPM7 channel and/or kinase are important in T cell development. Our TRPM7 kinase-dead mouse model, *Trpm7* ^*R/R*^, allows us to specifically address the function of TRPM7 kinase activity in T cells. The total numbers of thymocytes, as well as the percentages of double-negative (DN, CD4^−^CD8^−^), double-positive (DP, CD4^+^CD8^+^) and single-positive (SP, CD4^+^CD8^−^, CD4^−^CD8^+^) thymocytes were similar in both genotypes (Fig. 1a–c). Tissue-specific deletion of *Trpm7* in the T cell linage affected thymopoiesis through a block in the transition from the DN3 (CD25^+^CD44^−^) to the DN4 (CD25^−^CD44^−^) stage^18^. However, in the kinase-dead *Trpm7* ^*R/R*^ mutant, the distribution of DN3 and DN4 thymocytes was unaltered with respect to WT (Fig. 1d–f), indicating that the kinase activity is not responsible for the thymic phenotype observed previously.

In spite of normal T cell development and similar to T cell-specific conditional *Trpm7* ^*−/−*^ mice^18^, the *Trpm7* ^*R/R*^ mutant had a reduction of pro-inflammatory cytokines in the serum, including granulocyte colony-stimulating factor (G-CSF) and interleukin (IL)-17A. Also IL-1β, IL-3, IL-4, IL-9, IL-10, IL12p70, IL-13, granulocyte-macrophage colony-stimulating factor (GM-CSF), interferon (IFN)-γ and tumor necrosis factor (TNF) were reduced, albeit not significantly (Fig. 1g), thus indicating a function of the TRPM7 kinase in shaping the cytokine secretion profile.

In vitro activation of CD4^+^ T cells derived from *Trpm7* ^*R/R*^ mice using αCD3/αCD28-coated plates resulted in slightly reduced intracellular Ca^2+^ signalling compared to WT cells (Supplementary Fig. 2a). Although *Trpm7* ^*R/R*^ T cells had similar kinetics of receptor-operated Ca^2+^ entry (ROCE) compared to WT T cells, Ca^2+^ amplitudes in *Trpm7* ^*R/R*^ T cells were different at 150 s compared to WT (Supplementary Fig. 2a). Nonetheless, the proliferation rates were similar between the two genotypes, indicating no primary defect of *Trpm7* ^*R/R*^ mice in T cell activation (Supplementary Fig. 2b, c).

### TRPM7 kinase promotes T cell colonization of gut epithelium

**Fig. 2:**
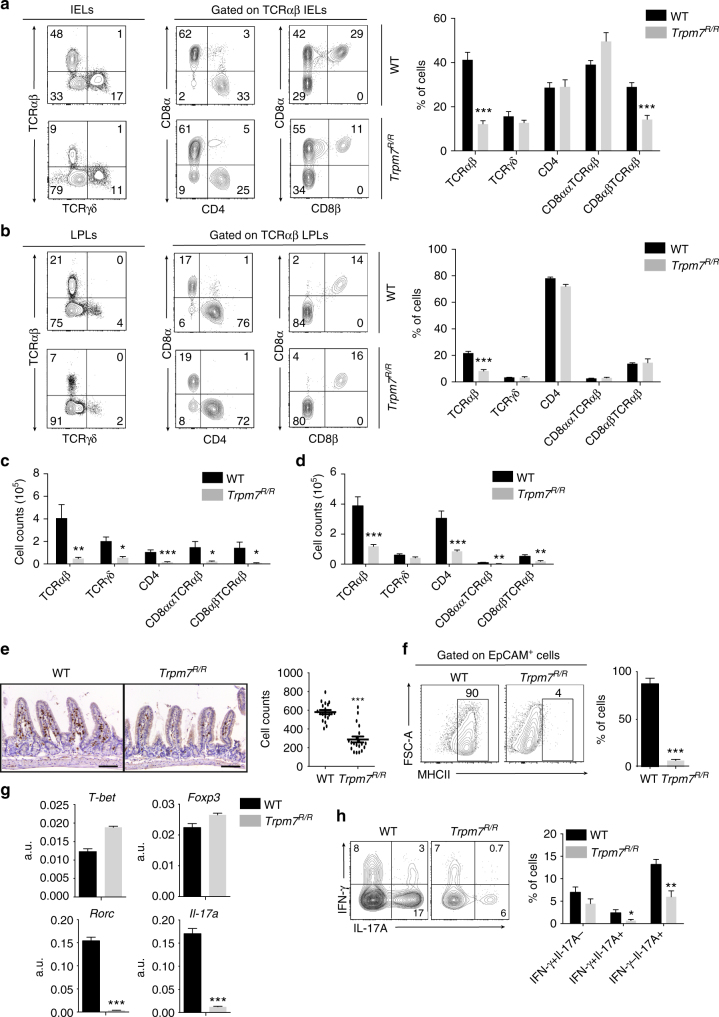
Selectively reduced intra-epithelial lymphocytes in *Trpm7* ^*R/R*^ mice. **a** Dot plot (left) and statistical analyses (right) of intra-epithelial lymphocytes (IEL) from WT or *Trpm7* ^*R/R*^ mice stained as indicated. Percentages are shown in each gate, bar charts show mean percentages ± s.e.m. (WT, *n* = 6; *Trpm7* ^*R/R*^, *n* = 7). **b** Dot plot (left) and statistical analyses (right) of lamina propria lymphocytes (LPL) from WT or *Trpm7* ^*R/R*^ mice stained as indicated. Percentages are shown in each gate, bar charts show mean percentages ± s.e.m. (*n* = 7). **c** Absolute numbers (WT, *n* = 6; *Trpm7* ^*R/R*^, *n* = 7) of the indicated IELs subsets. Bar charts show mean percentages ± s.e.m. **d** Absolute numbers (mean ± s.e.m. *n* = 7) of the indicated LPL subsets. **e** CD3 immunohistochemical staining of small intestine sections of WT or *Trpm7* ^*R/R*^ mice and relative quantification (right). Scale bars indicate 100 µm. **f** Dot blots and statistical analyses of MHCII expression in EpCAM^+^ intestinal epithelial cells (IEC). Percentages are shown in each gate, bar charts show mean percentages ± s.e.m. (*n* = 3). **g** Quantitative real-time PCR of *T-bet*, *Foxp3*, *Rorc* and *Il-17a* expression in purified TCRαβ^+^CD4^+^ IELs from WT or *Trpm7* ^*R/R*^ mice. **h** Dot plot and statistical analyses of IFN-γ and IL-17A staining in WT or *Trpm7* ^*R/R*^ TCRαβ^+^CD4^+^ IELs. Percentages are shown in each gate, bar charts show mean percentages ± s.e.m. (WT, *n* = 5; *Trpm7* ^*R/R*^, *n* = 8). Data are representative results of at least 3 independent experiments. A two-tailed Student’s *t* test was used with *p < 0.05; **p < 0.01 and ***p < 0.001

While T cell subsets in the spleen and peripheral lymph nodes were distributed normally in *Trpm7* ^*R/R*^ mice (Supplementary Fig. 3a, b), we found a strong reduction of all T cell subsets in the intestinal epithelium (Fig. 2a, c) and the lamina propria (LP) (Fig. 2b, d) by fluorescence-activated cell sorting (FACS) analysis. Notably, LPLs as well as CD4^+^ TCRαβ^+^ IELs were particularly affected by the lack of TRPM7 kinase activity (Fig. 2a, b). In line with these findings, the analysis of the distribution of CD3^+^ T cells in tissue sections of the small intestine from *Trpm7* ^*R/R*^ mice revealed a reduction of IELs compared to WT (Fig. 2e). The presence of IELs correlates with the induction of MHCII expression on epithelial cells^24^. Consistent with the reduction of IELs, we detected a dramatic reduction of MHCII expression in EpCAM^+^ intestinal epithelial cells in *Trpm7* ^*R/R*^ compared to WT mice (Fig. 2f). Analysis of the transcriptional profile of the few IELs that were present in *Trpm7* ^*R/R*^ mice revealed no differences in T-bet or FoxP3 expression when compared to WT, indicating a normal T_H_1 and T_reg_ polarization, respectively. However, the signature transcription factor for T_H_17 cells, Rorc, was reduced in *Trpm7* ^*R/R*^ IELs compared to WT that was also reflected by reduced IL-17 expression (Fig. 2g). These findings were confirmed by intracellular staining via FACS for IFN-γ and IL-17A in IELs isolated from WT and *Trpm7* ^*R/R*^ mice. While IFN-γ secreting cells were comparable between *Trpm7* ^*R/R*^ and WT IELs, IL-17A secreting cells were diminished in *Trpm7* ^*R/R*^ compared to WT IELs (Fig. 2h).

### Defect in gut epithelium colonization is T cell intrinsic

**Fig. 3:**
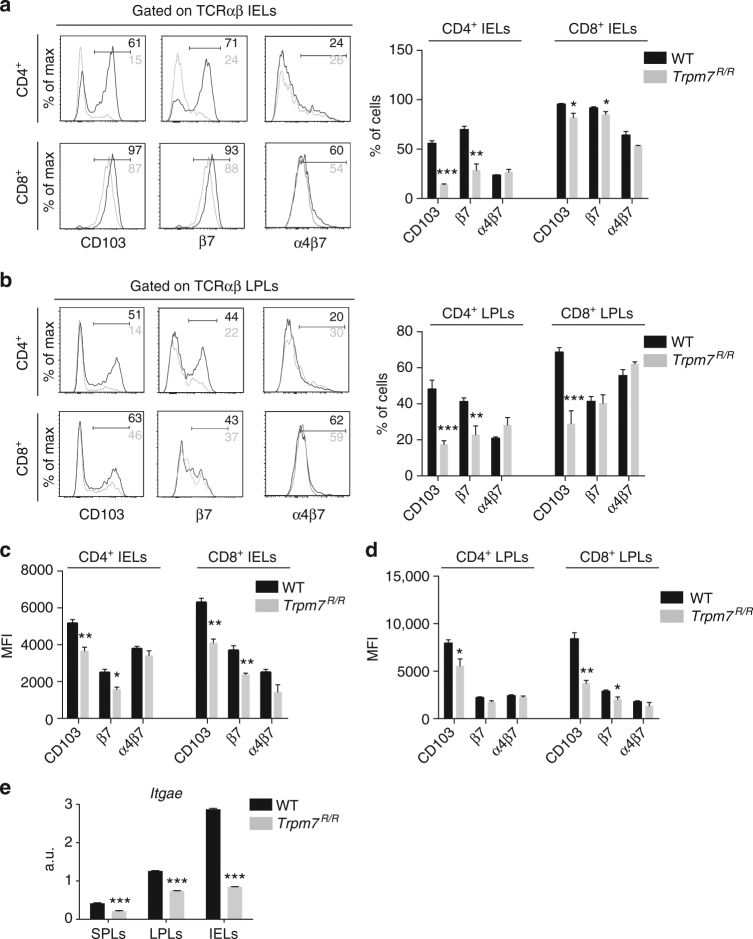
*Trpm7* ^*R/R*^ mutation affects α_E_β_7_ expression in T cells. **a** Representative histogram overlay of cell surface CD103, β_7_ and α_4_β_7_ expression of intra-epithelial lymphocytes (IEL, left) and relative statistical analysis (right). Percentages are shown in each gate, bar charts show mean percentages ± s.e.m. (*n* = 4). **b** Representative histogram overlay of cell surface CD103, β_7_ and α_4_β_7_ expression of lamina propria lymphocytes (LPL, left) and relative statistical analysis (right). Percentages are shown in each gate, bar charts show mean percentages ± s.e.m. (*n* = 4). **c** Surface CD103, β_7_, and α_4_β_7_ expression in IELs, bar charts show mean fluorescence intensity ± s.e.m. (*n* = 5). **d** Surface CD103, β_7_ and α_4_β_7_ expression of LPLs, bar charts show mean fluorescence intensity ± s.e.m. (*n* = 5). **e** Quantitative real-time PCR of *Itgae* expression in purified TCRαβ^+^CD4^+^ lymphocytes from spleen (SPL), lamina propria (LPL) or intra-epithelium (IEL). Data are representative results of at least 3 independent experiments. A two-tailed Student’s *t* test was used with *p < 0.05; **p < 0.01 and ***p < 0.001

In the intestinal epithelium the upregulation of CD103 is required, specifically integrin α_E_β_7_, which in turn interacts with E-cadherin on the epithelial cells and thus facilitates the retention of IELs into the epithelial layer^25, 26^. Interestingly, CD103 and integrin β_7_ expressing CD4^+^ IELs were reduced in *Trpm7* ^*R/R*^ mice, while CD8^+^ IELs were only slightly reduced and α_4_β_7_ expressing cells were unaffected (Fig. 3a). The analysis of CD4^+^ and CD8^+^ LPLs revealed a similar reduction in CD103 expression in *Trpm7* ^*R/R*^ mice compared to WT (Fig. 3b). However, integrin β_7_ expressing CD8^+^ LPLs were unaffected in *Trpm7* ^*R/R*^ mice compared to WT (Fig. 3b). Also the mean fluorescence intensity (MFI) of CD103 expression was reduced in *Trpm7* ^*R/R*^ CD4^+^ and CD8^+^ IELs as well as CD4^+^ and CD8^+^ LPLs compared to WT cells (Fig. 3c, d). Correspondingly, the MFI of the integrin β_7_ was similarly reduced (Fig. 3c, d). At the transcriptional level, analysis of the gene encoding CD103, *Itgae*, via quantitative real-time (qRT)-PCR revealed reduced *Itgae* messenger RNA (mRNA) expression in lymphocytes isolated from the spleen, LP and intestinal epithelium of *Trpm7* ^*R/R*^ compared to WT mice (Fig. 3e).

**Fig. 4:**
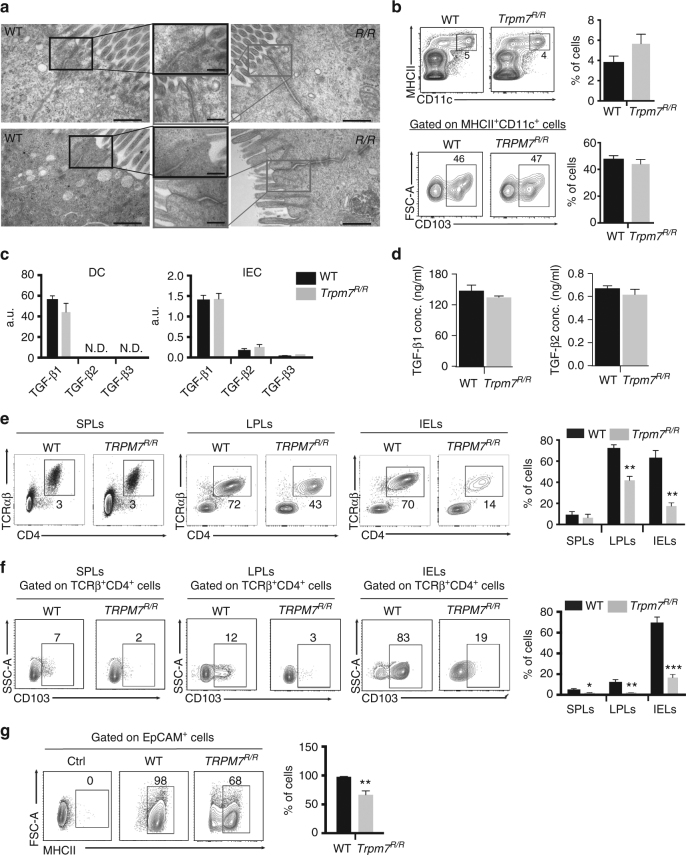
TRPM7 kinase-dead T cell autonomous defect in in vivo CD103 expression and intra-epithelial localization. **a** Transmission electron microscopic (TEM) images of small intestine (upper panel) and colon (lower panel) sections from WT or *Trpm7* ^*R/R*^ mice. Note no changes in tight junction, adherens junction or desmosome formation between the two genotypes. Scale bars indicate 500 and 200 nm, respectively. **b** Dot plot (left) and statistical analyses (right) of CD11c^+^MHCII^+^ DC and relative CD103 expression. Percentages are shown in each gate, bar charts show mean percentages ± s.e.m. (*n* = 3). **c** Quantitative real-time PCR of *Tgf-β1*, *Tgf-β2* and *Tgf-β3* expression in WT or *Trpm7* ^*R/R*^ purified CD11c^+^MHCII^+^ DC cells (left) or in EpCAM^+^ IEC (right). **d** TGF-β1 and TGF-β2 levels measured in serum harvested from WT or *Trpm7* ^*R/R*^ mice (*n* = 4). Data are shown as mean ± s.e.m. **e** Dot plot and statistical analyses of spleen (SPL), lamina propria (LPL) and intra-epithelial (IEL) TCRαβ^+^ CD4^+^ lymphocytes from *Rag1* ^−*/*−^ */Il2rg* ^−*/*−^ mice reconstituted with purified WT or *Trpm7* ^*R/R*^ naive CD4 cells. **f** Cells were gated for surface CD4 and TCRαβ and were analysed for CD103 expression. Percentages are shown in each gate, bar charts show mean percentages ± s.e.m. (*n* = 4). **g** Dot plots and statistical analyses of MHCII expression in EpCAM^+^ intestinal epithelial cells (IEC) from *Rag1* ^−*/*−^ */Il2rg* ^−*/*−^ mice reconstituted with purified WT or *Trpm7* ^*R/R*^ naive T cells. Percentages are shown in each gate, bar charts show mean percentages ± s.e.m. (*n* = 4). Data are representative results of at least three independent experiments. A two-tailed Student’s *t* test was used with *p < 0.05; **p < 0.01 and ***p < 0.001

To rule out the contribution of other cells to the reduction of IELs and LPLs as well as CD103 expression, we further examined intestinal epithelial as well as dendritic cells. Transmission electron microscopic images of the ileum (upper panel) and the colon (lower panel) of WT and *Trpm7* ^*R/R*^ mice illustrate no changes in overall structure, tight junction, adherens junction or desmosome formation (Fig. 4a), indicating no primary difference between the epithelial barrier of WT and *Trpm7* ^*R/R*^ mice. Interestingly, MHCII as well as CD103 surface expression of WT and *Trpm7* ^*R/R*^ dendritic cells was unaltered (Fig. 4b), suggesting that dendritic cell function is not affected by the TRPM7 kinase. Consistently, *Trpm7* mRNA levels were strongly reduced in DCs as well as in epithelial cells, compared to T cells (Supplementary Fig. 3c).

CD103 expression strongly depends on TGF-β stimulation^27^. The analysis of TGF-β1, two and three mRNA levels in dendritic as well as intestinal epithelial cells, two main sources of TGF-β in the gut, did not reveal significant differences between WT and *Trpm7* ^*R/R*^ mice (Fig. 4c). Moreover, we did not detect any difference in TGF-β serum levels between the different mice (Fig. 4d). Notably, TGF-β1 was the most prominent isoform in serum, while TGF-β3 was not detectable.

To confirm that the reduced number of IELs and LPLs in *Trpm7* ^*R/R*^ mice was T cell intrinsic, we adoptively transferred either WT or *Trpm7* ^*R/R*^ naive CD4^+^ cells into congenic *Rag1* ^*−/−*^ */Il2rg* ^−*/*−^ double mutant mice, lacking T and B as well as natural killer cells. While both WT and *Trpm7* ^*R/R*^ naive T cells equally reconstituted the spleen, *Trpm7* ^*R/R*^ T cells exhibited an intrinsic defect in colonizing the intestinal epithelium (Fig. 4e). *Trpm7* ^*R/R*^ CD4^+^ IELs poorly, if at all, expressed CD103 (Fig. 4f), thereby indicating that the defect of IEL retention within the small intestinal epithelium was T cell autonomous. Moreover, lymphopenic hosts adoptively transferred with naive CD4^+^ T cells from *Trpm7* ^*R/R*^ mice had impaired upregulation of MHCII in intestinal epithelial cells (Fig. 4g).

### TRPM7 kinase regulates TGF-β/SMAD pathways

**Fig. 5:**
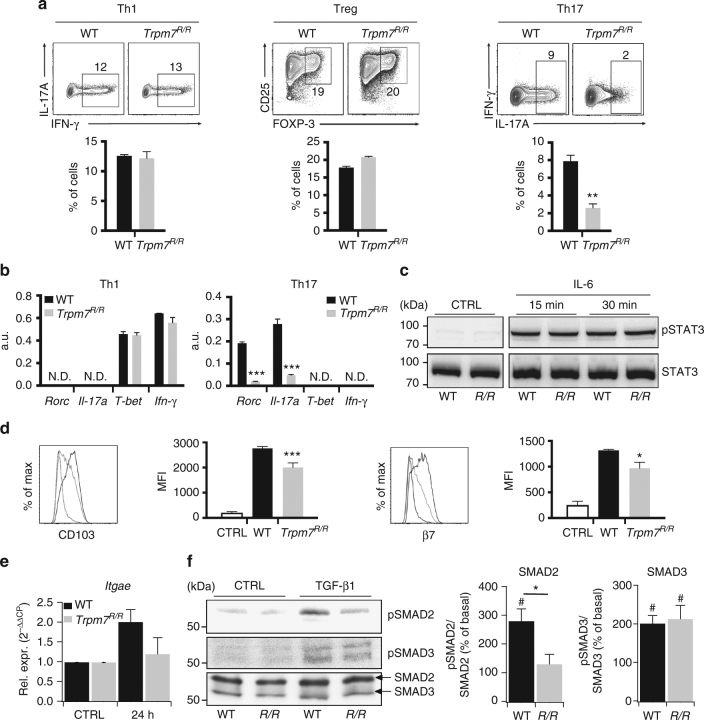
*Trpm7* ^*R/R*^ T cells have autonomous defects in in vitro Th17 polarization and CD103 upregulation. **a** Representative dot plots and statistical analyses of IFN-γ, IL-17A, CD25 and FOXP3 expression in stimulated naive T cells under Th1, T_reg_ or Th17-polarizing conditions after 5 days of in vitro culture. Percentages are shown in each gate, bar charts show mean percentages ± s.e.m. (*n* = 4). **b** Quantitative real-time PCR of *T-bet*, *ifn* *-γ*, *Rorc*, and *Il-17a* expression in naive T cells stimulated under Th1 or Th17-polarizing conditions after 5 days of culture in vitro. (*n* = 3). **c** Western blot analysis of STAT3 phosphorylation (Tyr705) of control and IL-6 treated WT and *Trpm7* ^*R/R*^ (*R/R*) naive T cells, respectively. Blots are representatives of at least three independent experiments. **d** Histogram overlays and statistical analyses of CD103 and β7 staining by flow cytometry in WT or *Trpm7* ^*R/R*^ naive T cells stimulated with anti-CD3ε/anti-CD28 in the absence or presence of TGF-β (10 ng ml^−1^) for 4 days. Histograms show mean fluorescence intensity (MFI) ± s.e.m. (*n* = 4). Data are representative results of at least three independent experiments. **e** Quantitative real-time PCR of *Itgae* (CD103) in control (CTRL) and WT or *Trpm7* ^*R/R*^ naive T cells stimulated with anti-CD3ε/anti-CD28 in the presence of TGF-β (5 ng ml^−1^) for 24 h. Data are shown as 2^−ΔΔCP^ ± s.e.m. (*n* = 3). **f** Western blot and statistical analysis of SMAD2 (Ser465/467) and SMAD3 (Ser423/425) phosphorylation. Blots are representatives of at least four independent experiments. The semi-quantitative analysis was done via ImageJ software and plotted as percent increase in intensity of pSMAD/total SMAD compared to control. Bar charts show mean percentages ± s.e.m. for SMAD2 and SMAD3 (*n* = 4–5). A two-tailed Student’s *t* test was used with *p < 0.05; **p < 0.01 and ***p < 0.001. To demonstrate a significant increase in TGF-β-induced SMAD phosphorylation compared to untreated controls a one-way ANOVA was used with ^#^ *p* < 0.05

As *Trpm7* ^*R/R*^ IELs displayed a pronounced reduction in Rorc and IL-17 expression while T-bet and FoxP3 were equivalent in *Trpm7* ^*R/R*^ compared to WT IELs (Fig. 2g), we addressed whether in vitro differentiation of naive CD4^+^ *Trpm7* ^*R/R*^ T cells would reproduce this phenomenon. After polarization of naive T cells into T_H_1 or T_reg_ for 5 days using the respective cytokine and inhibitory-antibody cocktails (Methods), we observed no differences in the percentage of IFN-γ or CD25^+^FoxP3^+^ T cells between the two genotypes (Fig. 5a, left and middle). Interestingly, in vitro polarization of naive CD4^+^ T cells into T_H_17 cells, using TGF-β, IL-6 and αIFN-γ, was reduced in *Trpm7* ^*R/R*^ compared to WT cells (Fig. 5a, right), consistent with the robust reduction of IL-17 concentration in serum from *Trpm7* ^*R/R*^ mice (Fig. 1g) as well as the diminished number of IL17-producing *Trpm7* ^*R/R*^ IELs (Fig. 2h). In contrast, *T-bet* and *Ifn-γ* mRNA levels were not different among in vitro-differentiated *Trpm7* ^*R/R*^ and WT T_H_1 cells (Fig. 5b). Since Rorc and IL-17 mRNA levels were reduced in in vitro-differentiated *Trpm7* ^*R/R*^ T_H_17 cells (Fig. 5b), we analysed STAT3 signalling as a signalling pathway involved in T_H_17 differentiation. However, western blot analysis of CD4^+^ T cells treated with IL-6 for 15 and 30 min showed no differences in STAT3 phosphorylation at Tyr705 (Fig. 5c).

**Fig. 6:**
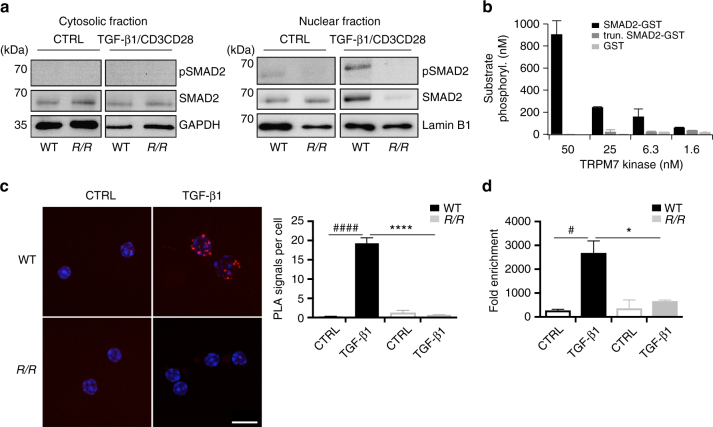
TRPM7 kinase affects SMAD2 translocation via direct phosphorylation. **a** Analysis of pSMAD2 translocation into the nucleus. WT and *Trpm7* ^*R/R*^ naive CD4^+^ T cells were co-stimulated with αCD3/αCD28 and 5 ng ml^−1^ TGF-β1 for 10 min. Representative western blot images depicting that pSMAD2 and total SMAD2 in the nuclear fraction (right) were strongly reduced in *Trpm7* ^*R/R*^ T cells compared to WT. In the respective cytosolic fraction (left), the pSMAD2 was not detectable, however amounts of total SMAD2 were comparable between *Trpm7* ^*R/R*^ and WT. **b** Concentration-dependent phosphorylation of human recombinant SMAD2-GST by TRPM7 kinase. Data have been obtained via RBC hotspot in vitro kinase assay using 4 µM ATP and 4 µM substrate at 2 h. RBC standard substrate was used as a positive control, substrate alone as a negative control and kinase activity alone was subtracted as background. Data have been converted to nM substrate phosphorylation and are plotted as mean ± s.e.m. Truncated recombinant SMAD2 (trun. SMAD2-GST) as well as the GST-tag alone were not phosphorylated, suggesting specific phosphorylation of SMAD2 at the c-terminal SXS motif. **c** Analysis of interaction between SMAD2 and TRPM7 in CD4^+^ T cells via proximity ligation assay (PLA). Scale bar indicates 10 µm. Note a significant increase in SMAD2 co-localization with TRPM7 in WT T cells treated with 5 ng ml^−1^ TGF-β1 (^####^ *p* < 0.0001; two-tailed Student’s *t* test). *Trpm7* ^*R/R*^ T cells fail to recruit SMAD2 into close proximity to the TRPM7 kinase upon TGF-β1 stimulation compared to WT (****p < 0.0001; two-tailed Student’s *t* test). Bar graphs show mean PLA signals per cell counted in five fields of vision ± s.e.m. **d** ChIP assay was performed in untreated and TGF-β1 (5 ng ml^−1^) stimulated CD4^+^ T cells using a specific antibody against SMAD2 for immunoprecipitation. Primers for *Itgae* and *Gapdh* were used for qRT-PCR; *Gapdh* was used for normalization. Note a significant increase in -fold enrichment in TGF-β1-treated WT T cells compared to untreated controls (^#^ *p* < 0.05, one-way analysis of variance) as well as a reduction in fold enrichment of TGF-β1-treated *Trpm7* ^*R/R*^ T cells compared to WT (*p < 0.05, one-way ANOVA). Bar graphs show mean ± s.e.m

Next, we asked whether the defect in CD103 expression in vivo was also reflected in vitro. To this end, naive CD4^+^ T cells were treated with TGF-β1, stimulated with αCD3/αCD28 and analysed for CD103 and integrin β_7_ surface expression by FACS. Interestingly, *Trpm7* ^*R/R*^ CD4^+^ T cells were characterized by a reduction in CD103 and integrin β_7_ expression (Fig. 5d). While WT naive CD4^+^ T cells increased *Itgae* gene expression twofold, *Trpm7* ^*R/R*^ naive CD4^+^ T cells were unable to upregulate *Itgae* expression after 24 h stimulation (Fig. 5e), suggesting a transcriptional regulation of CD103 via the TRPM7 kinase. Since TGF-β was shown to upregulate CD103 via SMAD and NFAT pathways in human T cells^28^, we addressed whether the TGF-β/SMAD signalling pathway was affected by TRPM7 kinase activity, particularly as TGF-β/SMAD pathways are also crucial for the polarization of CD4^+^ T cells into T_H_17 cells^29^. Importantly, western blot analysis of *Trpm7* ^*R/R*^ naive CD4^+^ T cells treated with 5 ng ml^−1^ TGF-β1 for 10 min revealed a strong and reduction in SMAD2 (Ser465/467) phosphorylation (Fig. 5f, upper row and middle panel), while SMAD3 (Ser423/425) phosphorylation was unaltered (Fig. 5f, middle row and right panel). Consistently, SMAD2 translocation into the nucleus was impaired in *Trpm7* ^*R/R*^ T cells compared to WT (Fig. 6a). Thus, we conclude that the TRPM7 kinase regulates T_H_17 differentiation and *Itgae* expression via TGF-β/SMAD2 dependent pathways.

To further clarify the mechanism by which TRPM7 kinase activity controls TGF-β/SMAD2 signalling, we performed an in vitro kinase assay using highly purified recombinant TRPM7 kinase, SMAD2-GST, as well as C-terminally truncated SMAD2-GST and GST-tag as controls. Remarkably, TRPM7 phosphorylates SMAD2 in a dose dependent manner. Moreover, TRPM7 fails to phosphorylate the truncated SMAD2 or the GST-tag, thereby identifying the C-terminal SXS motif of SMAD2 as a substrate for TRPM7 kinase (Fig. 6b). Thus, we conclude that TRPM7 kinase can modulate SMAD2 signalling via direct phosphorylation at the C-terminal Ser465/467 motif (Figs. 5f, 6b), which is essential for its transcriptional activity, while the linker region (Ser245/250/255) is unaffected by TRPM7 kinase (Supplementary Figs. 3d, 6b).

Moreover, we performed a proximity ligation assay (PLA) on purified CD4^+^ T cells, to characterize the interaction of SMAD2 with TRPM7 kinase in more detail. Figure 6c depicts a significant increase in SMAD2 co-localization with TRPM7 in WT T cells treated with 5 ng ml^−1^ TGF-β1 (*p* < 0.0001, two-tailed Student’s *t* test), while *Trpm7* ^*R/R*^ T cells fail to recruit SMAD2 into close proximity to TRPM7 kinase (Fig. 6c). SMAD2 has previously been shown to bind to the *Itgae* promoter sequence, thereby facilitating its transcription^25^. To link the observed defect in CD103 expression of *Trpm7* ^*R/R*^ T cells to their defective SMAD2 signalling, we performed a chromatin immunoprecipitation (ChIP) assay on primary murine CD4^+^ T cells with and without TGF-β1 stimulation (Fig. 6d). Our results show that SMAD2 binds to the *Itgae* promoter regions upon TGF-β1 stimulation in WT T cells, but fails to do so in *Trpm7* ^*R/R*^ T cells in response to TGF-β1 stimulation, underscoring the indispensable requirement of a functional TRPM7 kinase in TGF-β/SMAD2 signalling in T cells.

### TRPM7 kinase activity promotes graft-versus-host disease

**Fig. 7:**
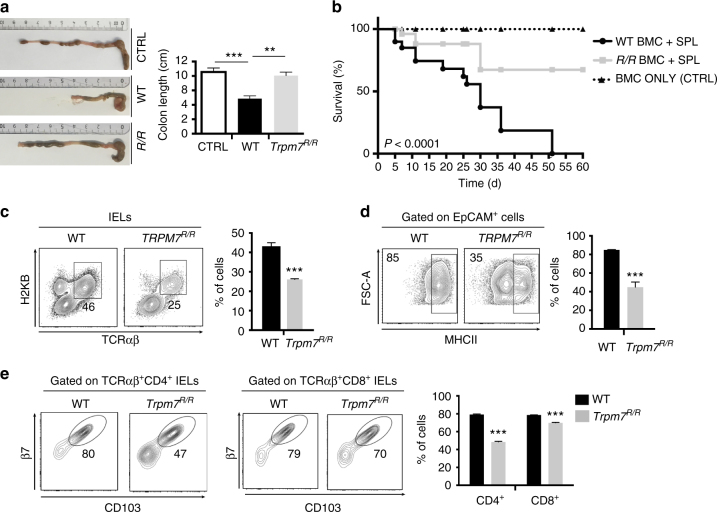
TRPM7 kinase activity promotes destruction of the host intestinal epithelium by T cells during GVHD. **a** Representative picture of colon specimens at day 25 after BMT in recipients of WT or *Trpm7* ^*R/R*^ splenocytes or (CTRL) bone marrow cells alone (left) and relative statistical analyses showing colon length (right). Bars represent mean ± s.e.m. (*n* = 5). **b** Survival of lethally irradiated BALB/c recipients of C57BL/6J bone marrow cells (BMC) alone (CTRL, triangle, dashed line) or in combination with WT (black circles) or *Trpm7* ^*R/R*^ (*R/R*, grey squares) splenocytes (*n* = 10). **c** Dot plot and statistical analyses of TCRαβ^+^H-2^b+^ IELs cells from BALB/c mice reconstituted with WT or *Trpm7* ^*R/R*^ splenocytes. Percentages are shown in each gate, bar charts show mean percentages ± s.e.m. (*n* = 3). **d** Dot plot and statistical analyses of MHCII expression in EpCAM^+^ IEC from BALB/c mice reconstituted with WT or *Trpm7* ^*R/R*^ splenocytes. Percentages are shown in each gate, bar charts show mean percentages ± s.e.m. (*n* = 3). **e** Dot plot and statistical analyses of CD103 and β7 expression in electronically gated H-2^b+^TCRαβ^+^CD4^+^ or H-2^b+^TCRαβ^+^CD8^+^ IELs. Percentages are shown within each gate, bar charts show mean percentages ± s.e.m. (*n* = 3)

In acute graft-versus-host disease (GVHD), naive donor CD4 cells recognize alloantigens on antigen presenting cells in target organs, including skin, intestine and lung. However, the function of different T_H_ subsets and signalling pathways in the pathogenesis of GVHD in distinct organs is incompletely characterized. We hypothesized that defective intestinal colonization by CD4^+^ cells lacking TRPM7 kinase activity could affect acute GVHD. To address this hypothesis, BALB/c WT mice were lethally irradiated and transplanted with bone marrow cells from WT C57BL/6J mice together with WT or *Trpm7* ^*R/R*^ splenocytes. As expected, injection of WT splenocytes resulted in massive intestinal damage as demonstrated by shortening of the colon (Fig. 7a) and most mice died within 35 days after transplantation (Fig. 7b). In contrast, injection of *Trpm7* ^*R/R*^ splenocytes did not cause intestinal damage and shortening of the colon in BALB/c hosts (Fig. 7a). Moreover, we observed a dramatically increased survival of these mice; only about 10% of mice injected with *Trpm7* ^*R/R*^ splenocytes died within the first 30 days after transplantation (Fig. 7b).

The analysis of intestinal epithelium by FACS with H2K^B^ (C57BL/6J haplotype)-specific mAb revealed a reduction of TCRαβ^+^ cells derived from *Trpm7* ^*R/R*^ splenocytes with respect to WT cells, suggesting an impairment of T cells lacking TRPM7 kinase activity in the colonization of host intestine (Fig. 7c). Also, the expression of CD103 and integrin β_7_ was reduced in CD4^+^ as well as CD8^+^ TCRαβ^+^ *Trpm7* ^*R/R*^ compared to WT cells (Fig. 7e). The reduction of gut colonization by *Trpm7* ^*R/R*^ T cells correlated with a reduced expression of MHCII in host intestinal epithelial cells with respect to mice injected with WT cells (Fig. 7d). These results indicate that TRPM7 kinase activity in T cells is a decisive factor in the pathogenesis of GVHD by promoting host gut epithelium colonization.

## Discussion

Tissue-specific deletion of *Trpm7* in the T cell lineage results in impairment of T cell development in the thymus and altered chemokine as well as cytokine expression profiles^18^. In contrast, mice carrying an inactive TRPM7 kinase (*Trpm7* ^*R/R*^) have unaltered thymopoiesis^21^, indicating that the channel but not the kinase activity is important in regulating the progression of T cell progenitors to mature T cells. However, in these mice, we observed a significant reduction of pro-inflammatory cytokines, including IL-17 and G-CSF, suggesting that TRPM7 kinase activity might be essential for immune system homoeostasis.

While T cells in the spleen and peripheral lymph nodes of *Trpm7* ^*R/R*^ mice were distributed normally, conventional T cells within IELs and LPLs were reduced. In particular, CD4^+^ T cells were the most significantly reduced IELs and LPLs subsets in *Trpm7* ^*R/R*^ as compared to WT mice. In addition, the analysis of functional subsets in the few CD4^+^ cells recovered from the gut of *Trpm7* ^*R/R*^ mice revealed a dramatic reduction of T_H_17 cells, indicating that TRPM7 kinase activity is important for gut colonization by T cells and T_H_17 cell differentiation. In fact, experiments of in vitro polarization of naive CD4^+^ T cells into T_H_1, T_reg_ and T_H_17 cells showed a selective defect of *Trpm7* ^*R/R*^ CD4^+^ T cells to polarize into Rorc and IL-17 expressing cells. STAT3 phosphorylation is important for T_H_17 cell differentiation^29^ and *Trpm7* silencing was shown to affect STAT3 phosphorylation at Tyr705 in breast cancer cells stimulated with epidermal growth factor^30^. However, IL-6 induced Tyr705 phosphorylation was unaffected in *Trpm7* ^*R/R*^ CD4^+^ T cells, suggesting that this signalling event is not involved in the defect in T_H_17 polarization of *Trpm7* ^*R/R*^ cells; this result also suggests that in breast cancer cells Tyr705 phosphorylation might be conditioned indirectly by the TRPM7 channel rather than kinase moiety.

In *Trpm7* ^*R/R*^ mice, the vascular adhesion molecule integrin α_4_β_7_ was not affected in intestinal T cells, whereas CD103 (integrin α_E_β_7_) was dramatically reduced. These data indicate that the profound reduction of intestinal T cells that characterizes these mice is due to the impaired retention of T cells mediated by the interaction of CD103 with E-cadherin expressed in epithelial cells rather than emigration from blood vessels into the LP^4^. Mice lacking CD103 have selectively reduced numbers of mucosal T cells and are more prone to experimentally induced colitis^25, 26^. However, this phenomenon was attributed to lack of CD103 in gut associated CD11c^high^MHCII^high^ dendritic cells (DCs)^31^, a cell population that was not affected by lack of TRPM7 kinase activity. Our observations are consistent with a selective defect of *Trpm7* ^*R/R*^ T cells in upregulating CD103 and gut retention, while CD103 expression is not affected in DCs by *Trpm7* ^*R/R*^, pointing to different regulatory mechanism/s in DCs. We demonstrated the T cell intrinsic nature of the intestinal defect due to lack of CD103 upregulation by adoptive transfer of *Trpm7* ^*R/R*^ CD4^+^ cells into lymphopenic hosts. Another important consequence of defective TRPM7 kinase activity in T cells was the significant reduction of MHCII expression in intestinal epithelial cells, a IELs-dependent feature essential for proper antigen presentation and immunological function of gut epithelial cells^1, 4^.

Both T_H_17-cell polarization and CD103 expression depend on TGF-β signalling^27, 28^. DCs and intestinal epithelial cells (IEL) are the major source for TGF-β in the gut^5^. However, the relative mRNA expressions of *Tgf-β1*, *2* and *3* in DCs and IELs as well as serum concentrations for TGF-β1 and 2 were similar both in *Trpm7* ^*R/R*^ and WT mice, indicating no primary defect in TGF-β production or secretion by lack of TRPM7 kinase activity. Conversely, in vitro induction of CD103 by TGF-β in naive *Trpm7* ^*R/R*^ CD4^+^ cells was impaired. This impairment was also evident at the transcriptional level since *Trpm7* ^*R/R*^ CD4^+^ cells failed to upregulate *Itgae*. In fact, according to SMAD dependence of *Itgae* expression^28^, we could show a reduction of the phosphorylation of the C-terminal SXS motif of SMAD2 but not of SMAD3 in TGF-β1-stimulated *Trpm7* ^*R/R*^ CD4^+^ cells. Moreover, using ChIP we demonstrated the defective binding of SMAD2 to the *Itgae* promoter region in *Trpm7* ^*R/R*^ T cells upon TGF-β1 stimulation. Interestingly, SMAD2 activation was suggested to exquisitely regulate T_H_17 cell generation but to be dispensable for T_reg_ cell differentiation^32^, consistent with distinct control of T cell functions by SMAD-dependent and -independent TGF-β signalling^33^. However, this notion remains controversial in the literature, as some studies report a dispensable function of SMAD2 in T_H_17 cell polarization^34–37^, suggesting the existence of compensatory mechanisms under certain circumstances. As we have not evaluated all possible Ser/Thr phosphorylation sites on SMAD3, we cannot exclude an effect of the TRPM7 kinase deletion on sites other than the C-terminal SXS motif. However, for SMAD2, we can exclude other direct phosphorylation sites, as the truncated SMAD2 mutant did not have any phosphorylation by TRPM7 kinase in our in vitro kinase assay. Nonetheless, our results are in line with a dispensable function of TRPM7 kinase activity in TGF-β mediated differentiation of CD4^+^ cells into T_reg_ cells.

TGF-β signalling exerts pleiotropic effects on cell physiology via cross-talk with multiple signalling pathways. Imaging of TGF-β1-activated SMAD signalling revealed selective inhibition of SMAD2 phosphorylation by distinct tyrosine kinase inhibitors^38^. TRPM7 kinase appears as a pharmacological target for inhibition of TGF-β1-mediated SMAD2 phosphorylation in T cells, as it is capable to directly phosphorylate SMAD2. In summary, our study demonstrates that TRPM7 kinase contributes to TGF-β-induced SMAD2 phosphorylation at Ser465/467 and translocation into the nucleus. Lack of TRPM7 kinase activity results in impaired transactivation of SMAD2 target genes, including *Itgae* (encoding for CD103), *Il-17* and *Rorc*, thus selectively limiting differentiation of the T cell along the T_H_17, but not T_reg_ cell, functional program. The protection of *Trpm7* ^*R/R*^ mice from GVHD, we have shown, unravels the clinical relevance of TRPM7 kinase as a target for limiting TGF-β-dependent CD103 expression as a pathogenetic mechanism in intestinal destruction during GVHD^27^. Finally, our study demonstrates the importance of developing pharmacological inhibitors for TRPM7 kinase activity to prevent the devastating consequences of acute GVHD without affecting the development of immunosuppressive T_reg_ cells.

## Methods

### Mice and in vivo experiments

*Trpm7* ^*R/R*^ mice were obtained from RIKEN, Japan^21^. Four- to eight-week-old male and female mice were used for all experiments. For ex vivo and in vitro experiments mice were killed using CO_2_ and terminated via cervical dislocation. All experiments involving animals at the Ludwig-Maximilians-Universität München, Munich, Germany were performed in accordance with the EU Animal Welfare Act and were approved by the District Government of Upper Bavaria, Germany, on animal care (permit no. 55.2-1-54^−^2532–134–13). The use of transgenic animals was approved by the District Government of Upper Bavaria, protocol no. 821–8763.14.718/1210. For in vivo experiments C57BL/6J, *Trpm7* ^*R/R*^, BALB/c and *Rag1* ^*−/−*^ */Il2rg* ^*−/−*^ mice were bred in a specific pathogen-free facility at the Institute for Research in Biomedicine, Bellinzona, Switzerland. For adoptive transfer of T naive, CD4^+^CD8^−^CD62L^+^CD44^−^CD25^−^ cells were sorted at FACSAria (BD Biosciences) from pooled cell suspensions of spleen, inguinal, axillary, brachial, cervical and mesenteric LNs of C57BL/6J and *Trpm7* ^*R/R*^ mice. Eight-week-old *Rag1* ^−*/*−^ */Il2rg* ^−*/*−^ mice were injected with 1 × 10^6^ naive T cells. Recipient mice were killed 4 weeks after reconstitution. For GVHD experiments, lethally irradiated (9 Gy, Cs source) BALB/c (H-2^d^) mice were reconstituted within 4–6 h by a single 0.2-ml intravenous inoculum containing 10 × 10^6^ B6 BMC alone or in combination with 10 × 10^6^ C57BL/6J or *Trpm7* ^*R/R*^ splenocytes. All animal experiments were performed in accordance with the Swiss Federal Veterinary Office guidelines and authorized by the Animal Studies Committee of Cantonal Veterinary with authorization numbers TI-10-2013 and TI-17-2015.

### Cell isolation and primary cell culture

Lymphocytes infiltrating the intestinal epithelium were isolated as follows: while the small intestine was flushed with PBS, fat and Peyer’s patches were removed. The small intestine was divided longitudinally, cut into 2-mm sections and washed twice, in calcium- and magnesium-free HBSS containing 2% fetal calf serum (FCS) (at 4 °C) to remove faeces. The tissue was placed in 50 ml tubes, washed three times in HBSS containing 2% FCS at 4 °C, transferred to 25 cm tissue culture flasks and incubated at 37 °C in HBSS containing 10% FCS, 0.2 mmol l^−1^ EDTA, 1 mmol l^−1^ DTT. After 20 min incubation, the flasks were shaken vigorously for 30 s, and the supernatant containing IELs and the IEC was separated from the tissue fragments using a 40-μm nylon filter. While the supernatant was collected and put on ice, the tissue fragments were retuned to the flasks and the process was repeated. To isolate LPLs, the remaining tissue was washed three times with RPMI 1640, and intestinal pieces were subsequently incubated with magnetic stirring for 30 min at 37 °C in cRPMI supplemented with 100 U ml^−1^ collagenase. The epithelial and lamina propria cell suspensions were washed, suspended in RPMI 1640 at 4 °C and filtered. The cell suspension was collected and suspended in 40% Percoll, which was layered on top of 80% Percoll and centrifuged at 2000 r.p.m. for 20 min at RT. The IELs and LPLs were collected from the interface between the Percoll gradients and prepared for phenotypic analysis by flow cytometry. For mRNA extraction, IELs and LPLs were purified by cell sorting as TCRβ^+^CD4^+^Ep-CAM^−^ cells while IEC cells were sorted as Ep-CAM^+^ cells. For isolation of thymocytes, thymi were homogenized and washed in RPMI1640 medium containing 10% (v/v) FBS. For the isolation of CD4^+^ T cells, peripheral lymph nodes were collected, smashed using a 40-μm strain and CD4^+^ T cells were sorted via magnetic-activated cell sorting (MACS) (CD4^+^ isolation kit, Miltenyi Biotec). Purity was assessed via FACS to at least 96% CD4^+^ T cells before cells were subjected to experiments. For mast cell isolation, cells obtained from the peritoneum of WT or *Trpm7* ^*R/R*^ mice were pelleted and apportioned (Cellgro) into Petri dishes with poly-D lysine (PDL)-coated glass cover slips. Cells were cultured in 2 ml DMEM containing 10% FBS (HyClone) and 1% penicillin/streptomycin (Gibco) overnight in a humidified incubator at 37 °C and 5% CO_2_. For electrophysiological experiments, mast cells were identified visually using light microscopy (phase contrast).

### Cytokine assays

After blood collection through cardiac puncture using a collector for serum separation and blood cells (Microvette, Sarstedt), samples were separated by 10.000×*g* centrifugation for 5 min; serum was then stored at −80 °C. Collected samples were prepared for the 23-cytokines assay (Bio-Rad) and TGFβ-1, 2, 3 assay (R&D Systems) according to manufacturer’s instructions.

### Antibodies and flow cytometric analysis

The following mAbs were purchased from BD Biosciences: allophycocyanin (APC)-conjugated anti-CD62l (clone:MEL-14, cat.#: 17-0621-83, working dilution 1:200), Pacific Blue-conjugated anti-CD8β (clone: H35-17.2, cat.#: 48-0083-80, working dilution 1:200), PERCP-CYANINE5.5-conjugated anti-IL-17A (clone: 17B7, cat.#: 45-7177-82, working dilution 1:100), fluorescein isothiocyanate (FITC)-conjugated anti-FOXP3 (clone: FJK-16s, cat.#: 11-5773-82, working dilution 1:100), Pacific Blue-conjugated anti-H-2KB (clone: AF6-88.5.5.3, cat.#: 48-5958-82, working dilution 1:200), APC-conjugated anti-CD11C (clone: N418, cat.#: 17-0114^−^82, working solution 1:200). The following mAbs were purchased from Biolegend (http://www.biolegend.com/): PE-conjugated anti-CD44 (clone:IM7, cat.#: 103008, working dilution 1:200), PE/Cy7-conjugated anti-CD25 (clone: PC61, cat.#: 102016 working dilution 1:200), APC/Cy7-conjugated anti-CD4 (clone: RM4-5, cat.#: 100526, working dilution 1:200), FITC-conjugated anti-CD8α (clone: 53-6.7, cat.# 100706, working dilution 1:200), PE-conjugated anti-TCRβ (clone: H57-597, cat.#: 109208, working dilution 1:200), FITC-conjugated anti-TCRγδ (clone: GL3, cat.#: 118106, working dilution 1:200), PE-conjugated anti-CD103 (clone: 2E7, cat.#: 121406, working dilution 1:200), PE-conjugated anti-α4β7 (clone: DATK32, cat.#: 120606, working dilution 1:200), APC-conjugated anti-β7 (clone: FIB504, cat.#: 321208, working dilution 1:200), Pacific Blue-conjugated anti-MHC-II (clone: M5/114.15.2, cat.#: 107620, working dilution 1:200), FITC-conjugated anti-Ep-CAM (clone: G8.8, cat.#: 118210, working dilution 1:200), PE-conjugated anti-IFN-γ (clone: XMG1.2, cat.#: 505808, working dilution 1:100). Samples were acquired on a LSRFortessa (BD Biosciences) or Guava (Merck-Millipore) flow cytometer. Data were analysed using FlowJo software (TreeStar, Ashland, OR), FACS Diva software (BD Biosciences) or InCyte (Merck-Millipore), respectively.

### Quantitative RT-PCR

Total RNA from FACS-sorted cells was precipitated in Trizol (Invitrogen, ThermoFisher) and reverse transcribed to complementary DNA (cDNA) using Random hexamers (Roche, cat.#: R 15504) and M-MLV reverse-transcriptase (Invitrogen, cat.#: 28025^−^013). For quantification of transcripts, mRNA samples were treated with 2 U per sample of DNase (Applied Biosystems). Transcripts were quantified by real-time PCR on an ABI PRISM 7700 Sequence Detector with predesigned TaqMan Gene Expression Assays and reagents according to the manufacturerʼs instructions (https://www.lifetechnologies.com). The following probes were used: *Trpm7* (Mm00457998_m1), *Tbx21* (Mm00450960_m1), *Foxp3* (Mm00475162_m1), *Rorc* (Mm01261022_m1), *Il17a* (Mm00439619_m1), *Itgae* (Mm00434443_m1), *Tgfβ1* (Mm01178820_m1), *Tgfβ2* (Mm00436955_m1), *Tgfβ3* (Mm01307950_m1). All reactions were performed in triplicates. The relative amounts of mRNAs were calculated by the ΔΔCT method. 18S and *Hprt* were used as internal housekeeping genes. For *Itgae* gene upregulation, CD4^+^ T cells were treated with 5 ng ml^−1^ of TGF-β1 (R&D) for 24 h. Total RNA was precipitated in Trizol (Invitrogen, ThermoFisher) and cDNA synthesis was performed using SuperScript II RT (LifeTech, Invitrogen) and oligo-dT primers (18T, Metabion). Real-time-PCR was performed using a PrimePCR^™^ SYBR^®^ Green Assay for *Itgae* (Bio-Rad, qMmuCID0039603) and analysed using LightCycler^®^ 480 SYBR Green I Master (Roche). *Hprt* (fwd: CTCATGGACTGATTATGGACAGG, rev: TTAATGTAATCCAGCAGGTCAGC, Metabion) was used as a reference gene. Samples were detected in doublets and the mean CP (crossing points) values were analysed as 2^−ΔΔCP^.

### In vitro T cell polarization and integrin upregulation

CD4^+^CD8^+^CD62L^+^CD44^−^CD25^−^ naive T cells were sorted at FACSAria from pooled suspensions of spleen, inguinal, axillary, brachial, cervical and mesenteric LNs of WT and *Trpm7* ^*R/R*^ mice. Cells were seeded in a 96-well, flat-bottomed plate in RPMI supplemented with 10% foetal calf serum (FCS) and 1% penicillin and streptomycin. For T cell in vitro polarization, Th1 cells were generated by addition of rmIL-12 at a concentration of 15 ng ml^−1^, hIL-2 30 U ml^−1^ and anti-IL-4 Ab (clone 11B11) at a concentration of 5 µg ml^−1^ into the culture. For the generation of Th17 cells naive T cells were cultured with rmIL-6 at a concentration of 20 ng ml^−1^, rmTGF-β at a concentration of 2 ng ml^−1^, anti-IFN-γ (clone XMG1.2) and anti-IL-4 Ab at a concentration of 5 µg ml^−1^. For the generation of T_reg_ cells, naive T cells were cultured with rmTGF-β at a concentration of 2 ng ml^−1^, 30 u ml^−1^ hIL-2, anti-IFN-γ and anti-IL-4 Ab at a concentration of 5 µg ml^−1^. For in vitro CD103 upregulation, T naive cells were stimulated in presence or absence of rmTGF-β at a concentration of 1 ng ml^−1^. After 4 days of stimulation, T cells were collected and stained with anti-CD103 and anti-β7 mAbs.

### Intracellular cytokine and transcription factor staining

For intracellular staining of FOXP3, after surface antigens staining, cells were fixed and permeabilized using the Foxp3/transcription factor staining buffer set (eBioscience) according to the manufacturer’s recommendations, followed by staining with anti-FOXP3. For intracellular staining of IFN-γ and IL-17A, cells were stimulated for 4 h with PMA (100 nM, Sigma-Aldrich) and ionomycin (1 μM, Sigma-Aldrich). Brefeldin A (BFA) was included during the last 4 h of activation to inhibit intracellular transport. After surface antigens staining cells were fixed and permeabilized using the BD Cytofix/cytoperm fixation/permeabilization solution Kit (BD Biosciences) according to the manufacturer’s recommendations, followed by staining with anti-IFN-γ and anti-IL-17A mAbs.

### Immunohistochemistry and digital image analysis

To assess the number of infiltrating T cells, 4 μm sections from each formalin-fixed paraffin embedded small intestinal sample were immunostained with a primary goat polyclonal antibody against CD3 epsilon antigen (Santa Cruz Biotechnology; #Sc-1127). A biotinylated rabbit anti-goat IgG antibody (BA-5000, Vector Laboratories, Burlingame, CA, USA) was added for 30 min and sections were then labelled by the avidin-biotin-peroxidase (ABC) procedure with a commercial immunoperoxidase kit (VECTASTAIN Elite ABC HRP Kit, PK-6100, Vector Laboratories, Burlingame, CA, USA). The immunoreaction was visualized with 3,3′-diaminobenzidine (peroxidase DAB substrate Kit, VC-SK-4100-KI01, Vector Laboratories, Burlingame, CA, USA) substrate and sections were counterstained with Mayer’s haematoxylin. For each sample, serial sections incubated with a 10% solution of normal rabbit serum served as negative controls. The number of CD3 epsilon^+^ cells and the area of the intestinal mucosa were evaluated using the ImageJ analysis program (http://rsb.info.nih.gov/ij/) in ×4200 microscopic fields. The number of T cells per mm^2^ of intestinal mucosa was then calculated.

### Transmission electron microscopy

Electron microscopy was preformed as follows: mice ileum and colon was washed with phosphate buffer (0.1 M; pH 7.2). Tissue was fixed in 2.5% glutaraldehyde in PB for 3 h, followed by washing the samples in phosphate buffer three times for 3 h. Samples were treated for 1.5 h with 1% osmium in H_2_O and increasing alcohol concentrations for dehydration. Finally samples were embedded in EPON^™^ and propylenoxid (propylenoxide: EPON^™^ = 3:1, 1:1, 1:3; 60 min each) followed by pure EPON^™^ for 2 days by 60 °C. Ultrathin sections were analysed in a Zeiss transmission electron microscope (EM902A).

### Western blot analysis

CD4^+^ T cells were seeded in 24-well plates and stimulated with 10 ng ml^−1^ IL-6 or 5 ng ml^−1^ TGF-β1 (PeproTech or R&D Systems) for the indicated time frames. For detection of phosphorylated proteins following antibodies were used: pSTAT3 (Tyr705, cat.#: 9131, Cell Signaling, molecular weight (MW) 86 kDa, working dilution 1:2500), pSMAD2 (Ser465/467, cat.#: 138D4, Cell Signaling, MW 60 kDa, working dilution 1:200) and pSMAD3 (Ser423/425, cat.#: C25A9, Cell Signaling, MW 52 kDa, working dilution 1:200). Total proteins were used as loading controls and stained for STAT3 (cat.#: 9132, Cell Signaling, MW 86 kDa, working dilution 1:5000) and SMAD2/3 (cat.#: D7G7, Cell Signaling, MW 60 kDa and 52 kDa, working dilution 1:1000). Cells were lysed with RIPA buffer. Lysates were subjected to SDS-PAGE, and proteins were transferred to nitrocellulose by western blotting. The first antibody was incubated overnight at 4 °C. After washing three times with TBS-T for 5 min, the membrane was incubated with a HRP-conjugated secondary antibody diluted in TBS-T and incubated for 45–60 min at RT. Immune reactivity was quantified by densitometry, ratios between p-SMAD2 or 3 and total SMAD2 or three signals, respectively, were calculated, and TGF-β1-induced SMAD phosphorylation was normalized to that of unstimulated cells. Data analysis was performed with the ImageJ analysis program (http://rsb.info.nih.gov/ij/). For analysis of the intensity of TGF-β1-induced SMAD phosphorylation compared to untreated controls a one-way ANOVA was used. Values of *p* < 0.05 (#) were considered significant. CD4^+^ T cells were seeded in 24-well plates and stimulated with 10 ng ml^−1^ IL-6, 5 ng ml^−1^ TGF-β1 (PeproTech or R&D Systems) and anti-CD3/anti-CD28-coated beads (Invitrogen) for 10 min^39^. For detection of phosphorylated proteins following antibody was used: pSMAD2 (Ser245/250/255, no. 3104, Cell Signaling, MW 60 kDa, working dilution 1:200). Total proteins were used as loading controls and stained for SMAD2 (D43B4, Cell Signaling, MW 60 kDa, working dilution 1:1000). Cells were lysed with RIPA buffer. Lysates were subjected to SDS-PAGE, and proteins were transferred to nitrocellulose by western blotting. The first antibody was incubated overnight at 4 °C. After washing three times with TBS-T for 5 min, the membrane was incubated with an HRP-conjugated secondary antibody diluted in TBS-T and incubated for 45−60 min at RT.

### In vitro kinase assay

Highly purified recombinant human SMAD2-GST, C-terminally truncated SMAD2-GST and GST were purchased from SignalChem (Richmond, BC, Canada, S11-30G-250, CUSTOM S11-30G-250, G52-30U-250). The in vitro kinase assay was performed by Reaction Biology Corp. (Woodbridge, CT, USA) following the RBC HotSpot Kinase Assay Protocol. RBC Standard reaction buffer contained: 20 mM Hepes (pH 7.5), 10 mM MgCl_2_, 1 mM EGTA, 2 nM MnCl_2_, 0.02% Brij35, 0.02 mg ml^−1^ BSA, 0.1 mM Na_3_VO_4_, 2 mM DTT, 1% DMSO. Reactions were carried out at 4 μM ATP in duplicates and measured at 1 h and 2 h, respectively. rhSMAD2-GST of 4 µM was used as substrate, and 4 µM rh-trSMAD2-GST as well as the 4 µM GST-tag alone were used as control substrates, while the TRPM7 kinase was titrated in a serial dilution starting at 50 nM. Kinase alone was subtracted as background. RBC standard substrate (MBP) was used as a positive and substrate alone as an additional negative control. Data acquired at 2 h were converted to nM substrate phosphorylation after background subtraction, averaged and plotted as mean values ± s.e.m.

### In situ proximity ligation assay

MACS-sorted CD4^+^ T cells from *TRPM7* ^*R/R*^ or WT mice were seeded on fibronectin coated cover slips (Carl Roth GmbH + Co. KG, cat.#: H873.2) in a six-well plate. After stimulation with 5 ng ml^−1^ TGF-β1 (R&D systems) for 10 min cells were fixed with 4% paraformaldehyde for 10 min and permeabilised with 0.2% Triton X-100 in PBS for 7 min. Blocking and the proximity ligation assay were performed with the DuoLink^®^ In situ Red Starter kit mouse/rabbit (Sigma-Aldrich, cat.#: DUO92101) according to the manufacturer’s instructions (http://www.sigmaaldrich.com/technical-documents/protocols/biology/duolink-fluorescence-user-manual.html). T cells were stained with anti-TRPM7 (self made, Dr. Chubanov, working dilution 1:100) and anti-SMAD2 (Santa Cruz, cat.#: sc-101153, working dilution 1:100) for 1 h at room temperature. DuoLink^®^ In situ PLA^®^ Probe anti-mouse PLUS and DuoLink^®^ In situ PLA^®^ Probe anti-rabbit MINUS were used for labelling anti-SMAD2 and anti-TRPM7 antibodies. Data acquisition was done on a Leica SP5 confocal microscope with a 63 × NA 1.4 PL APO objective (both Leica, Mannheim, Germany) by producing z-stacks of five randomly selected fields. Analysis of the data was done by production of maximum peak projections of the z-stacks and counting the PLA signals per cell manually. The mean number of PLA signals per cell was calculated per field. For comparison of two different sample groups, two-tailed unpaired Student’s *t* test was performed in Prism 6 (GraphPad Software, La Jolla, CA, USA).

### Chromatin immunoprecipitation

MACS-sorted CD4^+^ T cells from *Trpm7* ^*R/R*^ or WT mice were treated with or without 5 ng ml^−1^ TGF-β1 (R&D systems) for 10 min. In total, seven mice per genotype were used. Cells were cross-linked with 1% methanol-free formaldehyde and quenched with 0.125 M glycine. Nuclei were pelleted and lysed for 10 min on ice. After washings, lysates were sonicated four times for 30 s into DNA fragments of 200–2000 bp. Immunoprecipitation of the sheared chromatin was performed using an anti-SMAD2 (Cell Signaling Technology, cat.#: 5339 S.) antibody coupled to Dynabeads Protein G overnight at 4 °C. Sonicated chromatin of 1% was set aside as input without antibody. After washings of immune complexes and elution of DNA of both input and ChIP samples, qRT-PCR with specific primers for the *Itgae* (fwd: CCTCCACAGCCCTATGTGTT, rev: GCCTCACAGGTAGGAACTGG) and the *Gapdh* (fwd: CCCTGCTTATCCAGTCCTAGCTCA AGG, rev: CTCGGGAAGCAGCATTCAGGTCTCTGG) promoters for normalization was performed. For comparison of two different sample groups, one-way ANOVA was performed in Prism 6 (GraphPad Software, La Jolla, CA, USA).

### Determination of magnesium and calcium

Content of main elements in serum samples was determined by inductively coupled plasma mass spectrometry (ICP-MS) by ALS Scandinavia (Sweden). Therefore, serum was collected using a collector for serum separation and blood cells (Microvette, Sarstedt), samples were separated by 10.000×*g* centrifugation for 5 min; serum was then stored at −80 °C. Collected samples were shipped on dry ice for further analysis via ICP-MS.

### Immunoprecipitation and western blotting

Spleens were collected, smashed using a 100-μm strain, washed in PBS and subjected to red blood cell lysis. The red blood cell lysis buffer contained in mM: 160 NH4Cl, 10 KHCO3, 0.1 EDTA. After washing twice in PBS, splenocytes were lysed using a 1× lysis buffer containing: 0.5% (v/v), Igepal 0.5% (v/v), PMSF 1% (v/v), protease and phosphatase inhibitor 5 mM NaF. Lysates were incubated with a total TRPM7 antibody (ProScientifica, working dilution 1:50) and rotated for 2 h at 4 °C. Afterwards, Protein G sepharose beads (Dynabeads^®^, Invitrogen) equilibrated with lysis buffer were added at a working ratio 1:18 and rotated overnight at 4 °C. Immunoprecipitated lysates were subjected to SDS-PAGE, and proteins were transferred to nitrocellulose by western blotting. Following antibodies were used for detection: total TRPM7 (ProScientifica, working dilution 1:1000) pTRPM7Ser1511, working dilution 1:60). The first antibody was incubated overnight at 4 °C. After washing three times with TBS-T for 5 min, the membrane was incubated with a HRP-conjugated secondary antibody diluted in TBS-T and incubated for 45–60 min at R, and after subsequent washing steps, the chemiluminescent signal was detected.

### Generation of pTRPM7Ser1511-specific antibody

To generate a polyclonal pTRPM7Ser1511-specific antibody, rabbits were immunized with a phosphorylated peptide H2N-DSPEVD**(p)** SKAALLPC-NH2 coupled via its C-terminal cystein residue to keyhole limpet hemacyanin (phospho-peptide immunization program Eurogentec, Belgium). The generated serum was subjected to two rounds of peptide affinity chromatography. First, a fraction of antibody was purified using the phosphorylated peptide. Second, the isolated antibody was followed by an additional round of chromatography using a non-phosphorylated variant of the peptide (H2N-DSPEVDSKAALLPC-NH2) in order to deplete a fraction of antibody with cross-reactivity to a non-phosphorylated TRPM7. The final fraction of anti-pTRPM7Ser1511 antibody was aliquoted and stored at −80 ^o^C.

### ATP detection

Detection of ATP was performed using a conventional lucifern/luciferase assay, following manufacturer’s instructions (ATP Determination Kit, Invitrogen, Molecular Probes). Luminescence was monitored at ~560 nm using a microplate luminometer, FLUOstar OMEGA, by BMG.

### Electrophysiology

Patch-clamp experiments in whole-cell configuration were performed as follows: Currents were elicited by a ramp protocol from –100 mV to + 100 mV over 50 ms acquired at 0.5 Hz and a holding potential of 0 mV. Inward current amplitudes were extracted at –80 mV, outward currents at +80 mV and plotted versus time. Data were normalized to cell size as pA pF^−1^. Capacitance was measured using the automated capacitance cancellation function of the EPC-9/10 (HEKA, Lambrecht, Germany). Values over time were normalized to the cell size measured immediately after whole-cell break-in. Standard extracellular solution contained (in mM): 140 NaCl, 1 CaCl_2_, 2.8 KCl, 2 MgCl_2_, 10 HEPES-NaOH, 11 Gluc (pH 7.2, 300 mOsm). Nominally Mg^2+^-free extracellular solution contained (in mM): 140 NaCl, 3 CaCl_2_, 2.8 KCl, 10 HEPES-NaOH, 11 Gluc (pH 7.2, 300 mOsm). Divalent-free extracellular solution contained (in mM): 140 NaCl, 2.8 KCl, 10 HEPES-NaOH, 0.5 mM EDTA, 11 Gluc (pH 7.2, 300 mOsm). Standard intracellular solution contained (in mM): 120 Cs-glutamate, 8 NaCl, 10 HEPES, 10 Cs-EGTA, 5 EDTA (pH 7.2, 300 mOsm). For MgCl_2_ dose response intracellular solution contained (in mM): 120 Cs-glutamate, 8 NaCl, 10 Cs-BAPTA + appropriate amount of MgCl_2_ was added, as calculated with WebMaxC (http://www.stanford.edu).

### Calcium imaging

Intracellular calcium measurements were performed with freshly isolated naive CD4^+^ T cells. Measurements of intracellular Ca^2+^ levels with Fura-Red were made using dual excitation wavelengths of 420 and 470 nm (Invitrogen). CD4^+^ cells were loaded with 1 µM Fura-Red-AM in external solution for 30 min at room temperature. After incubation cells were centrifuged at 1.500 r.p.m. for 5 min at room temperature and resuspended in external solution containing (in mM) 140 NaCl, 2 CaCl_2_, 2.8 KCl, 1 MgCl_2_, 10 HEPES-NaOH, 11 Gluc (pH 7.2, 300 mOsm). Cells were transferred into a cell culture dish with glass bottom and kept in the dark at room temperature for 20 min. Then the dish was positioned in in the recording chamber. For basal Ca^2+^ concentrations, the mean of 5 ratio values recorded within the first minute after establishing a baseline was calculated. Images were analysed via the ZEN Software. Alternatively, naive CD4^+^ T cells were loaded with 2 µM Fura-2-AM, 1% BSA and 0.02% Pluronic^®^ F-127 in external solution for 15 min at room temperature in the dark. Cells were transferred into a cell culture dish with glass bottom, and stimulated with plate-bound anti-CD3ε and anti-CD28 (5 and 2 µg ml^−1^, respectively). Images were analysed with TILLvisION software.

### In vitro T cell proliferation

CD4^+^ naive T cells were seeded in a 96-well, flat-bottomed plate in RPMI supplemented with 10% FCS and 1 % penicillin and streptomycin. In proliferation assays, cells were labelled with the ThermoFisher CellTrace violet (#C34557) and stimulated by plate-bound anti-CD3ε (2 μg ml^−1^) mAb with or without co-immobilized anti-CD28 mAb (2 μg ml^−1^) (eBioscence). CellTrace dilution was measured in truly live cells through the exclusion of dead cells by electronic gate of Propidium Iodide negative cells. FACS acquisitions were standardized by fixed numbers of calibration beads (BD Biosciences). Alternatively, 0.5 × 10^6^ CD4^+^ T cells per ml were seeded into 96-round-bottom-well plates coated with anti-CD3 (5 µg ml^−1^) as well as anti-CD28 (5 μg ml^−1^). Every day cells were resuspended in medium and 50 μl were analysed via FACS analysis (Guava, Merck-Millipore) using the ViaCount dye (Merck-Millipore) to count live cells.

### Statistical analysis

Unless stated otherwise, a two-tailed unpaired Student’s *t* test was used to determine the significance of differences between mean values (GraphPad or IgorPro). Data are presented as mean values ± s.e.m. of at least three mice. Values of *p* < 0.05 were considered significant with *p < 0.05, **p < 0.01 and ***p < 0.001.

### Data availability

The authors declare that the data supporting the findings of this study are available within the paper and its supplementary information file.

## Electronic supplementary material

Supplementary Information
