# Supplementary material for: TRPM7 kinase activity is essential for T cell colonization and alloreactivity in the gut
Source: Nat Commun. 2017 Dec 4;8:1917. doi: 10.1038/s41467-017-01960-z (PMC5714948; doi:10.1038/s41467-017-01960-z)
Supplement: Supplementary file 1 — Supplementary Information [file 41467_2017_1960_MOESM1_ESM.pdf]

Supplementary Information

Supplementary Figures and Figure Legends
Supplementary Western Blot Images
Supplementary Table

Supplementary Figures and Figure Legends

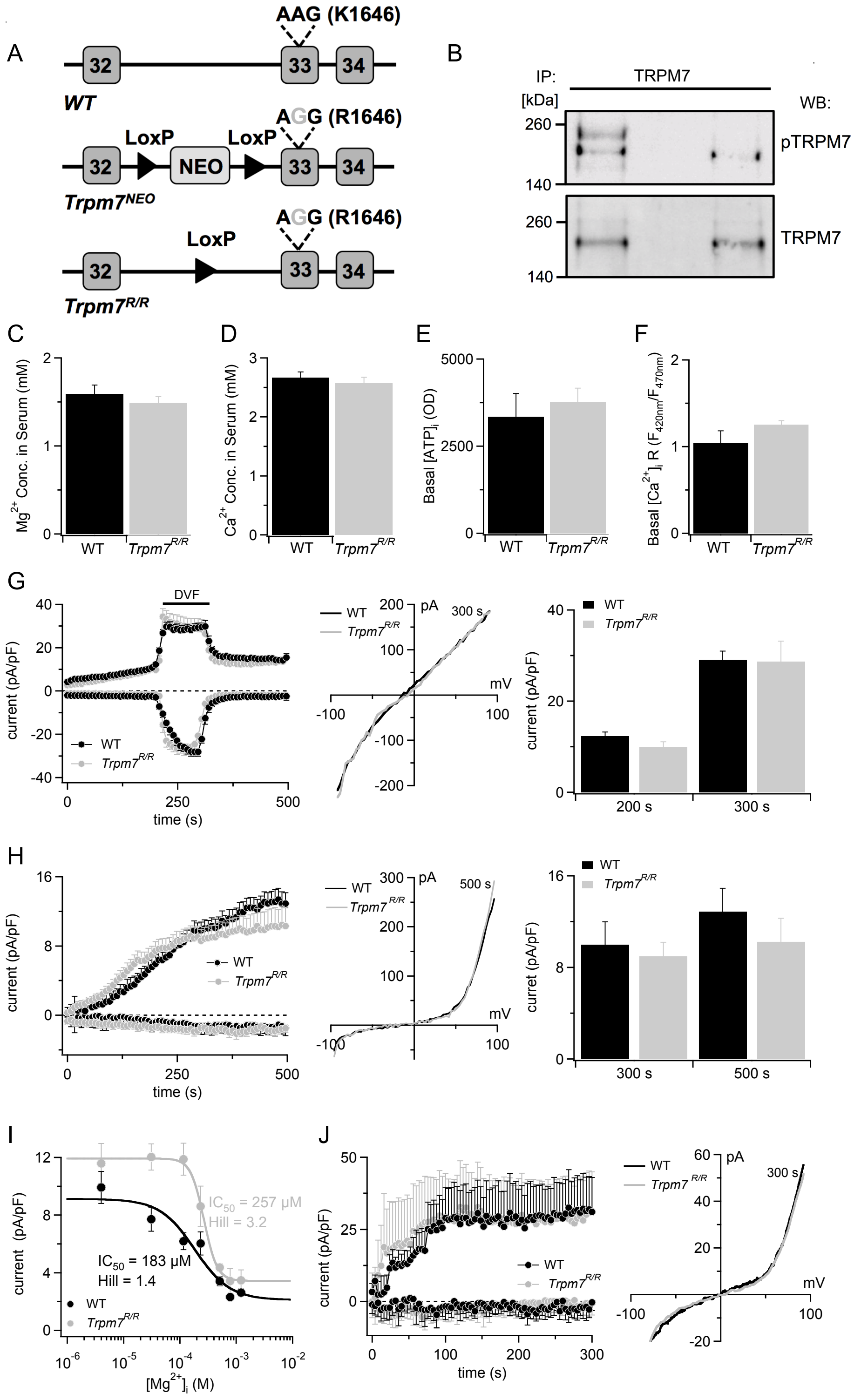

Supplementary Figure 1

Phenotype of *Trpm7$^{R/R}$* mutant mice (**a**) Targeting strategy for the generation of *Trpm7$^{R/R}$* mutant mice. (**b**) Immuno-precipitation (IP) and Western Blot (WB) analysis of primary splenocytes isolated from WT or *Trpm7$^{R/R}$* mice, respectively. (**c,d**) Mg^{2+} and Ca^{2+} concentrations in WT and *Trpm7$^{R/R}$* sera. Ions in the serum of individual mice were measured with inductively coupled plasma mass spectrometry (ICP-MS). Data are shown as average concentrations in mM (WT: black, Mg: n = 7, Ca: n = 13 and *Trpm7$^{R/R}$*: grey, Mg: n = 8, Ca: n = 14). Error bars indicate s.e.m. (**e**) Bar graph showing averages of luciferase luminescence detecting ATP levels in cell lysates of CD4^{+} T cells. Averages of

WT (black, n = 4) and *Trpm7*$^{R/R}$ (grey, n = 4) are shown and error bars indicate s.e.m. (**f**) Bar graphs comparing average Fura-Red ratios of WT (black, n = 28) and *Trpm7*$^{R/R}$ (grey, n = 31) $CD4^{+}$ T cells indicating cellular, free Ca^{2+} concentrations at rest. Error bars indicate s.e.m. (G-J) Electrophysiological characterization of TRPM7 in WT and *Trpm7*$^{R/R}$ derived immune cells. Whole-cell currents were recorded in freshly isolated primary peritoneal mast cells, or $CD4^{+}$ T lymphocytes. Voltage ramps were elicited from −100 to +100 mV over 50 ms, acquired at 0.5 Hz and recorded at an interval of 2 s. TRPM7 current amplitudes were assessed at +80 mV for outward currents and at -80 mV for inward currents, averaged and normalized to cell size (pF). Error bars indicate s.e.m. (**g**) Whole-cell patch clamp analysis of TRPM7 current development in WT (black, n = 6) and *Trpm7*$^{R/R}$ (grey, n = 8) mast cells. In the left panel the current densities at +80 and -80 mV are plotted versus time of the experiment in seconds (s). Representative current-voltage relationships extracted at 500 seconds of WT (black) and *Trpm7*$^{R/R}$ (grey) cells are shown in the middle panel. Bar graphs of current densities at +80 mV extracted at 300 and 500 seconds (right panel). Measurements have been conducted in absence of extracellular and intracellular Mg^{2+} to elicit maximal TRPM7 currents. (**h**) To estimate functional channel expression, we perfused WT (black, n = 12) and *Trpm7*$^{R/R}$ (grey, n = 9) mast cells with divalent-free solution (DVF, left panel). Current densities were plotted *versus* time of the experiment. Representative current-voltage relationships extracted at 300 s (middle panel). Bar graphs of mean current densities at +80mV extracted at 200 s and 300 s. WT (black, n = 5-12) and *Trpm7*$^{R/R}$ (grey, n = 6-12). (**i**) Mg^{2+}-dose-response curve of outward current densities plotted against different $[Mg^{2+}]_i$ concentrations. (**j**) Experiments and analysis were performed as in (**g,h**), using, WT (black, n = 5) and *Trpm7*$^{R/R}$ (grey, n = 5) primary, murine $CD4^{+}$ T cells.

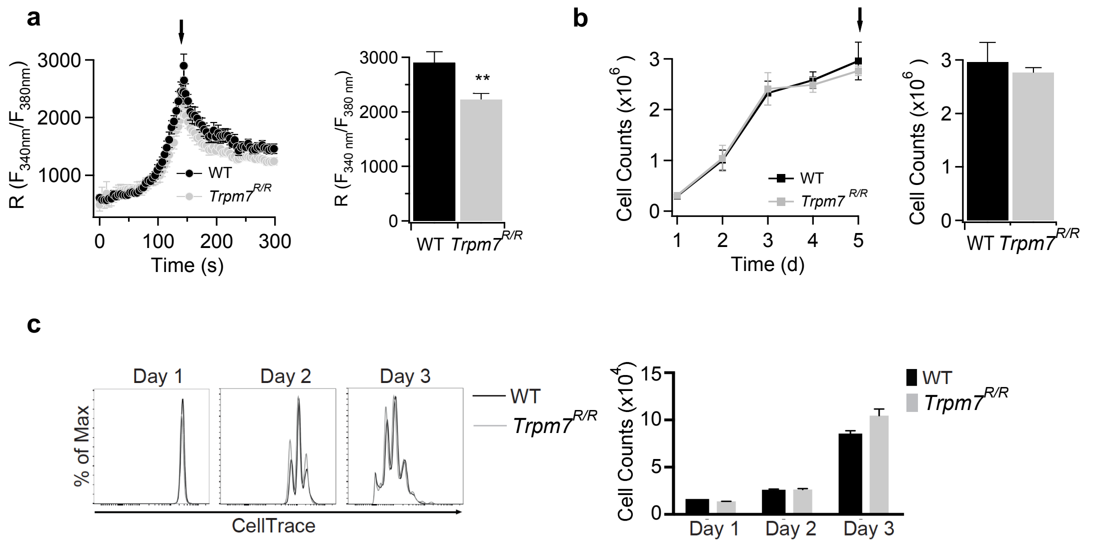

Supplementary Figure 2

Ca^{2+} signaling and proliferation of $CD4^{+}$ T cells. (**a**) Ratiometric Ca^{2+} measurements of WT (black, n = 28) and *Trpm7*$^{R/R}$ (grey, n = 31) $CD4^{+}$ T cells stimulated with αCD3/αCD28 were plotted over time (s). In total 3 WT and 3 *Trpm7*$^{R/R}$ mice were used. A bar graph extracted at 150 s shows mean values ± s.e.m. (right panel). (**b**) T cell proliferation shown in cell numbers plotted *versus* time (days). $CD4^{+}$ T cells isolated from WT (black, n = 3-4) and *Trpm7*$^{R/R}$ (grey, n = 3-4) were stimulated with αCD3/αCD28 antibodies. A total number of 5 animals were used each. In the right panel data at day 5 are shown as average cell numbers ± s.e.m. (**c**) Representative overlay (left) showing the CellTrace profile of WT or *Trpm7*$^{R/R}$ naïve T cells stimulated for 3 days with αCD3/αCD28 and statistical analysis (right) of the total number of recovered cells at the indicated day. Data are representative results of at least 3 independent experiments. * $p<0.05$; ** $p<0.01$ and *** $p<0.001$.

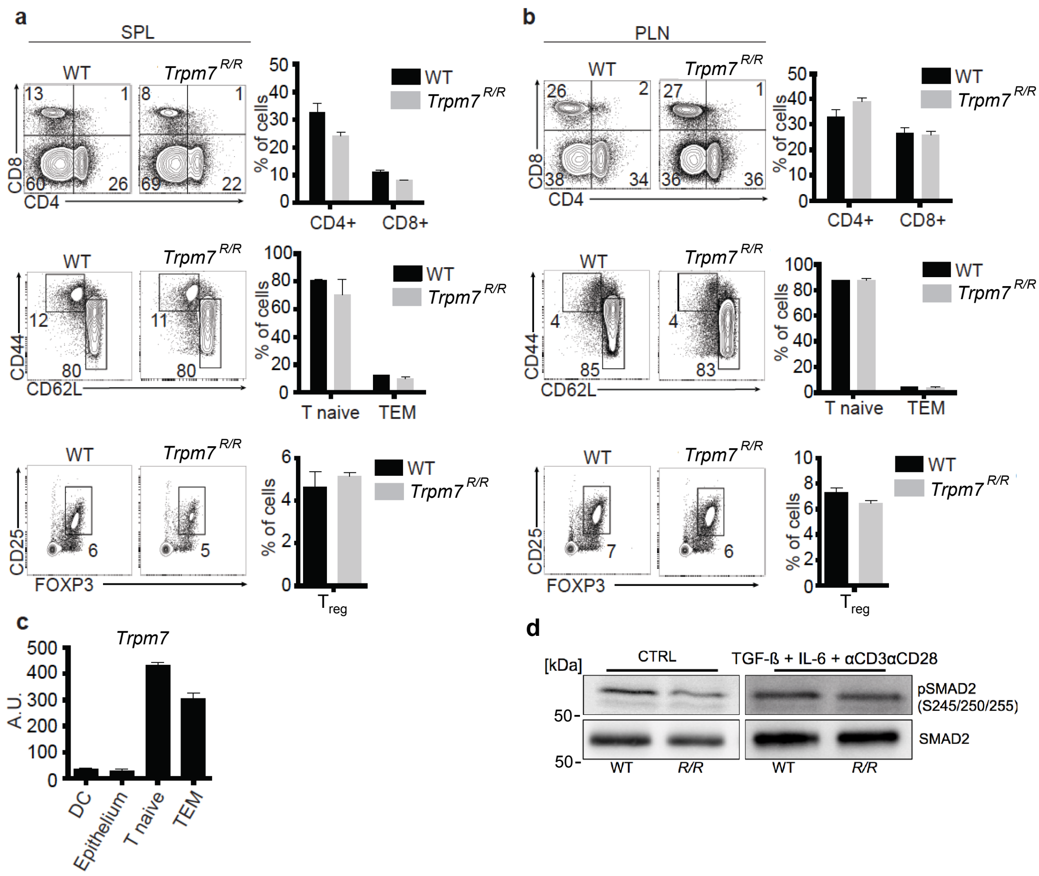

Supplementary Figure 3

Lymphocyte distribution in peripheral lymphoid organs and TRPM7 expression. (**a**) Dot plot and statistical analyses of lymphocytes derived from spleen (SPL) of WT or *Trpm7*$^{R/R}$ mice stained as indicated. Percentages are shown in each gate, histograms show mean percentages ± s.e.m. (WT, n=5; *Trpm7*$^{R/R}$, n=5). (**b**) Dot plot and statistical analyses of lymphocytes from peripheral lymph nodes (PLN) of WT or *Trpm7*$^{R/R}$ mice stained as indicated. Percentages are shown in each gate, histograms show mean percentages ± s.e.m. (WT, n=5; *Trpm7*$^{R/R}$, n=5). Data are representative results of at least 3 independent experiments. (**c**) Quantitative real-time PCR of TRPM7 expression in WT purified CD11c^{+}MHCII^{+} dendritic cells (DC), EpCAM^{+} epithelial cells (EC), naïve T cells as well as effector memory T cells (TEM) (n=3). Note that T cells express substantially more TRPM7 transcripts compared to DC or EC. (**d**) Representative Western Blot analysis of SMAD2 phosphorylation at the linker region (Ser245/250/255) induced via co-stimulation of naïve T cells with 5 ng ml^{-1} TGF-β1, 10 ng ml^{-1} IL-6 and αCD3/αCD28 for 10 min.

Supplementary Full Blots

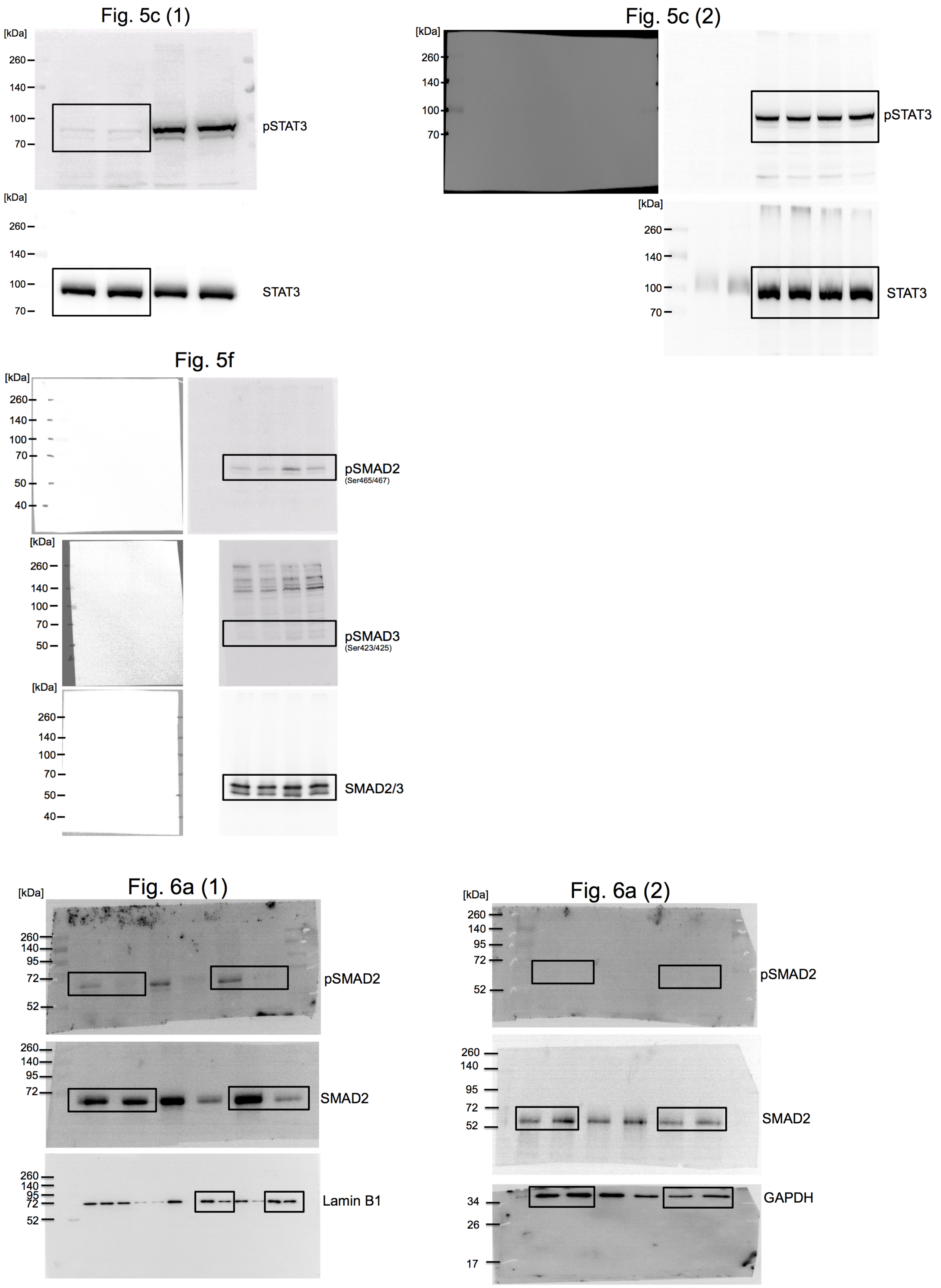

Supplementary Figure 4
Original data of immunoblots of Figures 3, 5 and 6.

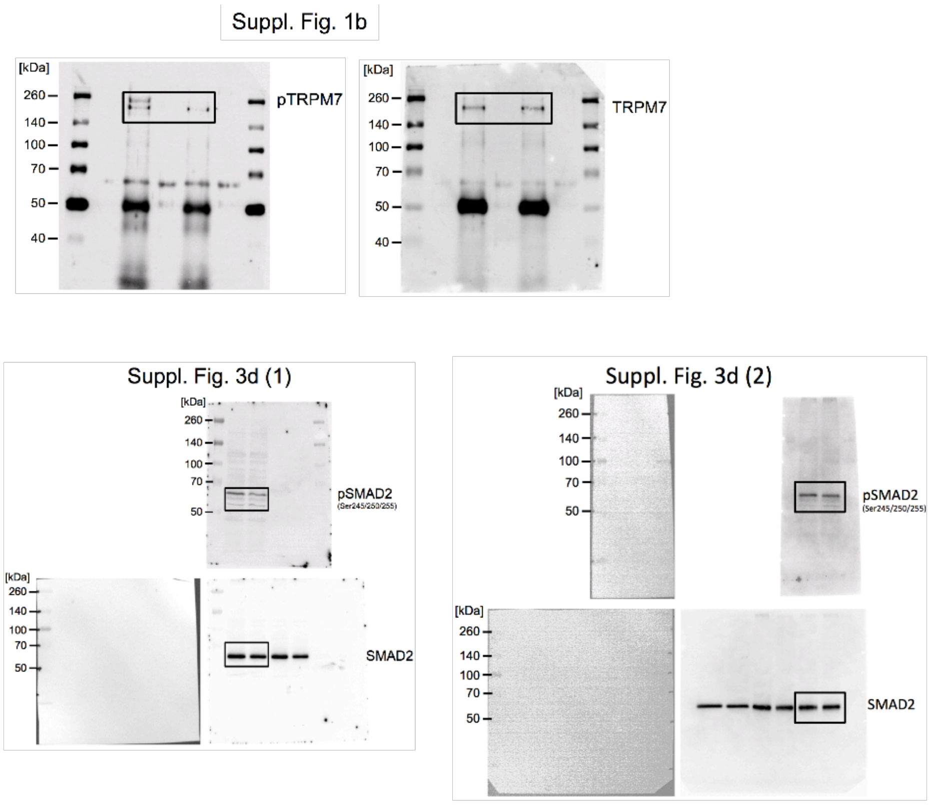

Supplementary Figure 5
Original data of immunoblots of Supplementary Figures 1 and 3.

Supplementary Table

Gene Name	species	Oligonucleotide sequence or catalogue number
Trpm7	mouse	Assay ID: Mm00457998_m1
Tbx21	mouse	Assay ID: Mm00450960_m1
Foxp3	mouse	Assay ID: Mm00475162_m1
Rorc	mouse	Assay ID: Mm01261022_m1
Il17a	mouse	Assay ID: Mm00439619_m1
Itgae	mouse	Assay ID: Mm00434443_m1
Itgae	mouse	Assay ID: qMmuCID0039603
Itgae	mouse	Fwd: CCTCCACAGCCCTATGTGTT<br>Rev: GCCTCACAGGTAGGAACTGG
Tgfβ1	mouse	Assay ID: Mm01178820_m1
Tgfβ2	mouse	Assay ID: Mm00436955_m1
Tgfβ3	mouse	Assay ID: Mm01307950_m1
Hprt	mouse	Fwd: CTCATGGACTGATTATGGACAGG,<br>Rev: TTAATGTAATCCAGCAGGTCAGC
Gapdh	mouse	Fwd:CCCTGCTTATCCAGTCCTAGCTCAAGG<br>Rev:CTCGGGAAGCAGCATTCAGGTCTCTGG

Supplementary Table 1
List of all oligonucleotide sequences or catalogue numbers used in qRT-PCR or ChIP-PCR.
